# Epicardial adipose tissue mediates the association between circulating hsa-miR-4750-3p and coronary artery disease in patients with type 2 diabetes mellitus

**DOI:** 10.1186/s12933-025-03055-2

**Published:** 2026-01-15

**Authors:** Joanna Szydełko, Tomasz Zapolski, Monika Lenart-Lipińska, Marcin Czop, Alicja Petniak, Janusz Kocki, Beata Matyjaszek-Matuszek

**Affiliations:** 1 Department of Endocrinology, Diabetology and Metabolic Diseases, Medical University of Lublin, Jaczewskiego 8, Lublin, 20-090 Poland; 2 Department of Cardiology, Medical University of Lublin, Jaczewskiego 8, Lublin, 20-090 Poland; 3 Department of Clinical Genetics, Medical University of Lublin, Radziwillowska 11, Lublin, 20-080 Poland

**Keywords:** Epicardial adipose tissue, miRNA, miR-4505, miR-4743-5p, miR-4750-3p, Type 2 diabetes mellitus, Coronary artery disease

## Abstract

**Background:**

Epicardial adipose tissue (EAT) is a metabolically active visceral fat depot contributing to coronary atherosclerosis, yet the molecular mechanisms underlying EAT-related coronary artery disease (CAD) in type 2 diabetes mellitus (T2DM) remain unclear. Previously, we identified novel circulating miRNAs targeting fatty acid metabolism in T2DM-CAD. This study aimed to investigate whether EAT may explain the association between dysregulated hsa-miR-4505, hsa-miR-4743-5p, hsa-miR-4750-3p and CAD in T2DM patients and whether it can detect diabetic atherosclerosis alone or in a multi-modal combination.

**Methods:**

Seventy-six patients with T2DM and/or CAD along with eighteen healthy controls were enrolled in the study. All participants underwent transthoracic echocardiography to assess EAT thickness on the free wall of the right ventricle at end-systole and bioelectrical impedance analysis for body composition determination. Spearman’s rank correlation and multivariate linear regression accounting for relevant clinical confounders were used to explore the associations between EAT and miRNAs. To further investigate whether EAT acts as an intermediary between miRNA and CAD in T2DM, causal mediation analysis was employed. The receiver operating characteristics curves were generated to evaluate the diagnostic performance of the combined models built using multivariate logistic regression.

**Results:**

The median EAT thickness was significantly higher in T2DM-CAD patients compared to T2DM subjects and controls (*p* < 0.0001). The bivariate analysis showed a positive correlation between triglyceride concentration and EAT thickness, and a negative one with hsa-miR-4750-3p expression. After multivariable adjustment, hsa-miR-4750-3p (*β* = − 0.445, *p* = 0.003) emerged as a standalone predictor of EAT thickness. Logistic regression analysis identified enlarged EAT, up-regulated hsa-miR-4505, hsa-miR-4743-5p and down-regulated hsa-miR-4750-3p to be independently associated with higher CAD risk in T2DM. Adding miRNAs to EAT improved CAD detection in T2DM (AUC = 0.988), outperforming both EAT (AUC = 0.869), clinical factors (AUC = 0.829), and their combination (AUC = 0.901). The mediation analysis revealed that EAT accounted for 48.79% of the total effect of hsa-miR-4750-3p on CAD in T2DM.

**Conclusions:**

These findings suggest that the proposed miRNA-EAT regulatory axis may be involved in the pathogenesis of diabetic atherosclerosis, with EAT appearing to partially mediate the relationship between hsa-miR-4750-3p and CAD. The integrated approach linking EAT and miRNAs holds potential for CAD risk stratification in T2DM.

**Supplementary Information:**

The online version contains supplementary material available at 10.1186/s12933-025-03055-2.

## Background

 Type 2 diabetes mellitus (T2DM) and coronary artery disease (CAD) are the leading causes of morbidity and mortality, with an ever-increasing prevalence globally [1, 2]. Despite advancements in the diagnostic and therapeutic strategies, patients with T2DM are still at a two- to four-fold increased risk of cardiovascular diseases compared to those without diabetes [3]. In recent years, visceral adipose tissue and, in particular, cardiac fat depots have gained a special attention as novel independent predictors of cardiometabolic risk in individuals with T2DM [4, 5].

Epicardial adipose tissue (EAT) is a metabolically active and unique subset of visceral fat surrounding the major epicardial coronary arteries and myocardium [6]. Due to its unobstructed proximity to the heart, and the absence of fascial boundaries, EAT shares the same microcirculation with the myocardium, allowing for bi-directional paracrine and vasocrine crosstalk between adipocytes, cardiomyocytes, and vascular wall cells [6, 7]. Epicardial adipocytes have a greater capacity for the release, uptake and storage of free fatty acids (FFAs) and a lower rate of glucose utilization compared to other visceral fat deposits, functioning as a buffer to protect the myocardium from lipotoxicity [8]. However, the cardioprotective role of EAT may be compromised under pathological conditions, such as triglyceride overload or hypoxia [9, 10]. Consequently, EAT tends to switch to a pro-inflammatory phenotype that alters vascular homeostasis and endothelial cell function by increased release of pro-inflammatory cytokines, FFAs and decreased secretion of anti-inflammatory adipokines, promoting the occurrence and progression of atherosclerosis [6, 8].

Since epicardial adipocytes have revealed a specific transcriptome and secretome profile, circulating non-coding RNAs have attracted growing interest due to their putative modulatory properties in EAT-mediated cardiac effects [11, 12]. MicroRNAs (miRNAs, miRs) are small, single-stranded non-coding RNAs that posttranscriptionally regulate the expression of critical genes, either by guiding target mRNA degradation or inducing translational repression [13]. Of note, miRNAs released into extracellular fluids through mechanisms involving lipid vesicle trafficking and protein/lipoprotein carriers may act as autocrine, paracrine, and endocrine messengers in cell-to-cell communication [13, 14]. Thus, packaged circulating miRNAs become resistant to RNase-dependent degradation, ensuring their stability and effective delivery to distant recipient cells, enabling functional gene regulation and modulation of cellular activity [14–16]. Importantly, these properties also render circulating miRNAs highly attractive non-invasive disease biomarkers [17]. Owing to their ability to target multiple genes and jointly regulate individual transcripts, circulating miRNAs represent candidate epigenetic regulators in complex pathways related to metabolic homeostasis, adipogenesis, inflammation, and endothelial dysfunction [12, 17].

In our previous study, we identified a plasma three-miRNA signature, comprising up-regulated hsa-miR-4505 and hsa-miR-4743-5p and down-regulated hsa-miR-4750-3p, which was able to effectively distinguish T2DM-CAD patients from those with T2DM better than each single miRNA alone [18]. Upon bioinformatics analysis, we unveiled that the gene targets of all three miRNAs were overrepresented in processes related to fatty acid metabolism [18]. Therefore, we hypothesized that our newly discovered miRNAs might be associated with EAT dysfunction.

Emerging evidence suggests that epicardial fat is an important link coupling diabetes and cardiovascular diseases [19]. The latest meta-analyses demonstrated abnormal thickening of EAT in both CAD and diabetic patients compared to non-CAD and non-diabetic subjects, regardless of the measurement method used [20–22]. Moreover, it has been observed that the miRNA expression profile and secretory function of epicardial adipocytes are altered under hyperglycemic conditions, as well as during atherogenesis [23–25]. Although much is known about the role of EAT and miRNAs in the pathogenesis of various cardiometabolic diseases, the interaction of miRNAs with EAT-related CAD in T2DM remains unexplored.

To address this gap, the present study aimed to investigate the association of echocardiographic EAT thickness with selected clinical indices and circulating hsa-miR-4505, hsa-miR-4743-5p, hsa-miR-4750-3p, particularly focusing on the mediating effect of EAT on the interplay between miRNA and CAD among patients with T2DM. Secondarily, we sought to elucidate the potential role of EAT, when used alone or in a multi-modal combination, in predicting CAD in T2DM.

## Methods

### Study design and patients

This single-center, observational, case-control study was conducted in the Department of Endocrinology, Diabetology and Metabolic Diseases at the Medical University of Lublin, Poland, between October 2020 and December 2022. A cohort of 76 Caucasian participants aged 45–65 years with T2DM and/or CAD (T2DM group, *n* = 30; T2DM-CAD group, *n* = 30; CAD group, *n* = 16) was enrolled in the study, along with 18 healthy controls matched for age, sex, and body mass index (BMI). The recruitment of patients was based on the rigorous eligibility criteria, as reported in the previously published paper [18].

Briefly, the diagnosis of T2DM was established according to the American Diabetes Association guidelines [26]. Patients with T2DM and/or CAD underwent invasive coronary angiography which was assessed by two independent interventional cardiologists, and CAD was defined as at least one major epicardial vessel with > 50% stenosis, while those without any stenosis or luminal irregularities in any of the epicardial arteries were classified as CAD negative ones [2]. The control subjects were randomly selected from healthy volunteers who had normal glucose tolerance and no evidence of CAD.

In the present study, we excluded patients with other types of diabetes mellitus, micro- and other macrovascular or acute diabetic complications, autoimmune diseases, acute/chronic inflammatory diseases, human immunodeficiency virus or hepatitis C virus infection, liver and renal dysfunction, cancers, poor echocardiographic image quality, the presence of 1–50% diameter stenosis in the epicardial coronary artery on coronary angiography, previous myocardial infarction, percutaneous coronary intervention and/or coronary artery bypass grafting, heart failure, cardiac arrhythmias, moderate to severe valvular heart disease, congenital heart disease, and cardiomyopathy. Moreover, patients who were addicted to smoking, reported a daily alcohol consumption greater than or equal to 30 g for men and 20 g for women, underwent surgery or trauma in the past 3 months, or used drugs with the proven effect on blood counts in the past 6 months were eliminated from the investigation. All subjects had a stable body weight for the past 6 months and were on a stable hypoglycemic, antihypertensive, lipid-lowering, and/or antiplatelet therapy for at least 3 months prior to study inclusion.

The study was approved by the Bioethics Committee of the Medical University of Lublin, Poland (No. KE-0254/198/2020) and conducted in accordance with Good Clinical Practice (Declaration of Helsinki of 1975, revised in 2013). Participation in the study was voluntary, and all patients provided written informed consent.

### Anthropometric and clinical measurements

All measurements were performed after an overnight fast, with participants in a standing position, wearing light clothing and barefoot. Body weight was measured using a digital electronic scale (to the nearest 0.1 kg), and height was assessed with a portable stadiometer (to the nearest 0.1 cm). BMI was calculated as weight in kilograms divided by height in meters squared, and obesity was defined as a BMI of 30.0 kg/m^2^ or greater. Waist circumference (WC) was taken at the midpoint between the anterior superior iliac spine and the lower edge of the rib cage using a plastic, non-elastic measuring tape at the end of a normal expiration, and hip circumference (HC) was measured at the widest diameter over the greater trochanters (both to the nearest 0.1 cm). Waist-to-hip ratio (WHR) as the ratio of waist circumference to hip circumference was determined.

Body composition, including fat mass (FM), fat-free mass (FFM), total body water (TBW), and visceral fat rating (VFR), was evaluated using bioelectrical impedance analysis (TANITA DC-430 S MA, Tanita Corporation, Tokyo, Japan) following the manufacturer’s recommendations.

Systolic (SBP) and diastolic (DBP) blood pressure were taken in three sets using an oscillometric method in a sitting position after a minimum of 5 min of quiet rest, and calculated as the average of the second and third measurements.

### Biochemical measurements

Whole blood (10 mL) was collected by venipuncture between 7:30 and 8:30 a.m. after an overnight fast into plastic tubes (S-Monovette, Sarstedt, Nümbrecht, Germany) and processed within 30 min to obtain plasma and serum for routine biochemical analyses, as described earlier [18]. Plasma glucose, glycated hemoglobin A1c (HbA1c) and fibrinogen, serum triglycerides (TG), total cholesterol (TC), high-density lipoprotein (HDL)-C, uric acid, creatinine, high-sensitivity C-reactive protein (hs-CRP), interleukin-6 (IL-6), and total homocysteine concentrations were quantified by automated standard methods in a centralized laboratory using Atellica CH 930 analyzer (Siemens Healthineers, Erlangen, Germany). Low-density lipoprotein (LDL)-C concentration was estimated using the Friedewald equation. Estimated glomerular filtration rate (eGFR) was calculated according to the Modification of Diet in Renal Disease (MDRD) formula.

Complete blood counts were analyzed using an automated hematology analyzer (Yumizen H500 Analyzer, Horiba Medical, Northampton, UK). Indirect inflammatory indices, such as neutrophil-to-lymphocyte ratio (NLR), monocyte-to-lymphocyte ratio (MLR), and platelet-to-lymphocyte ratio (PLR), were calculated by dividing the absolute neutrophil, monocyte, and platelet counts by the absolute lymphocyte count, respectively.

### Total RNA isolation and reverse transcription quantitative real-time polymerase chain reaction

The detailed protocol of total RNA isolation and reverse transcription quantitative real-time polymerase chain reaction (RT-qPCR) to determine the expression of three miRNAs (hsa-miR-4505, hsa-miR-4743-5p, hsa-miR-4750-3p) in plasma, selected by our group based on miRNA profiling with microarrays and bioinformatic analysis, was presented in our previously published paper [18]. All the PCR reactions were run in triplicate. The data were analyzed with Expression Suite Software version 1.0.3. (Applied Biosystems, Thermo Fisher Scientific, Waltham, MA, USA). The relative quantification (RQ) of miRNA expression, after normalization to small nuclear RNA (snRNA) U6 as an endogenous control, was calculated using the 2^−ΔΔCt^ method, and logarithmically transformed to reduce skewness [27].

### Standard echocardiography

Two-dimensional (2D) transthoracic echocardiography (TTE) was performed on each participant using commercially available equipment (Sonos 7500, Philips, Andover, MA, USA) with a 2.5–3.5 MHz transducer in accordance with the recommendations of the American Society of Echocardiography and the European Association of Cardiovascular Imaging [28]. All measurements were taken by an experienced and certified cardiac sonographer (T.Z.) who was blinded to the patients’ clinical data. Standard parasternal and apical two- and four-chamber views were obtained with the patients in the left lateral decubitus position. All patients were in sinus rhythm at the time of echocardiographic examination.

The left ventricular (LV) end-diastolic (EDD) and end-systolic (ESD) diameters were measured in the parasternal long-axis view at the level of the mitral valve leaflet tips, and the LV fractional shortening (FS) was determined by the formula (LVEDD-LVESD)/LVEDD × 100%. The LV end-diastolic (EDV) and end-systolic (ESV) volumes were measured in the apical two- and four-chamber views using the biplane method of discs summation (modified Simpson’s rule), and the LV ejection fraction (EF) was calculated according to the following equation [(LVEDV-LVESV)/LVEDV] × 100% [28].

### Measurement of epicardial adipose tissue thickness

**Fig. 1:**
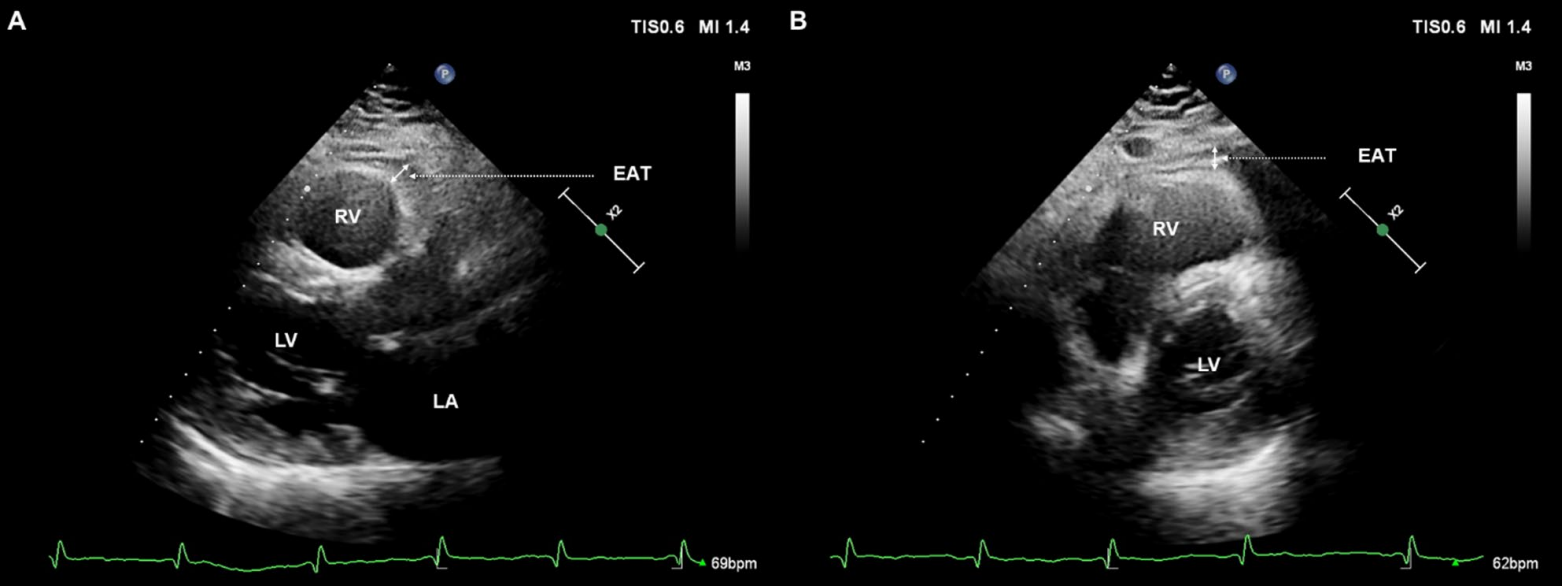
Echocardiographic measurement of EAT thickness on the free wall of the RV at end-systole. **A** Parasternal long-axis view. **B** Parasternal short-axis view. EAT thickness (white solid line) was identified as the echo-free space between the outer wall of the myocardium and the visceral layer of the pericardium. EAT, epicardial adipose tissue; RV, right ventricle; LV, left ventricle; LA, left atrium

EAT was identified as the echo-free space between the outer wall of the myocardium and the visceral layer of the pericardium on 2D TTE. EAT thickness was measured perpendicularly on the free wall of the right ventricle (RV) at end-systole in the parasternal long- and short-axis views, as first proposed and validated by Iacobellis et al. [29, 30]. Maximum EAT thickness was measured from the parasternal long-axis images at the point on the free wall of the RV along the midline of the ultrasound beam, perpendicular to the aortic annulus, used as an anatomical landmark for this view (Fig. 1A) [30, 31]. For the midventricular parasternal short-axis assessment, maximum EAT thickness was measured on the RV free wall along the midline of the ultrasound beam perpendicular to the interventricular septum at midchordal and tip of the papillary muscle level, as anatomic landmarks (Fig. 1B) [30, 31]. The average value of three cardiac cycles from each echocardiographic view was considered for analysis.

All measurements of EAT thickness were performed by a single experienced echocardiographer (T.Z.). Intraobserver and interobserver variability were assessed in 20 randomly selected patients. Intraobserver variability was evaluated by comparing two measurements of EAT thickness conducted by the same cardiac sonographer (T.Z.) with an interval of one week. Interobserver variability was assessed by comparing measurements independently performed by two observers (T.Z. and J.S.). Both observers were blinded to the patients’ clinical data and to each other’s results to avoid bias.

### Statistical analysis

Categorical variables were expressed as numbers with percentages (%) and compared using the χ2 test. Continuous variables were presented as mean ± standard deviation (SD) or median with interquartile range (IQR). The Shapiro–Wilk test was applied to assess the normality of the data distribution. Comparisons between normally distributed variables were performed by Student’s *t*-test for two groups or one-way analysis of variance (ANOVA) with Tukey’s post hoc test for unequal *n* for more than 2 groups. The Mann–Whitney *U* test or the Kruskal-Wallis test followed by Dunn’s test were utilized to compare data with non-Gaussian distribution between two or more than two groups, respectively.

A Bland–Altman analysis was performed to assess the agreement between EAT thickness measured in the parasternal long- and short-axis views [32]. The bias was calculated as the mean of the differences in the results obtained from the different projections, while the 95% limits of agreement were estimated as the mean difference ± 1.96 times the standard deviation of the differences [32]. To calculate the correlation between the two methods of measuring EAT, Lin’s concordance correlation coefficient was applied. The intra- and interobserver variability of EAT thickness measurements was evaluated using the intraclass correlation coefficients (ICCs) based on two-way mixed- and random-effects models with an absolute agreement [33].

Correlation coefficients between EAT thickness, miRNA expression, and selected clinical parameters were analyzed using the Spearman’s rank correlation. Candidate variables with *p*-values of less than 0.10 in univariate analysis and without collinearity, along with those pre-specified based on clinical relevance, were included in multivariate linear regression to identify independent determinants of EAT thickness. A variance inflation factor above 5 was set as the exclusion criterion to avoid multicollinearity between parameters.

Univariate and multivariate binary logistic regression analyses were performed to identify variables that were significantly and independently associated with the occurrence of CAD in T2DM. The results were presented with odds ratios (ORs) and 95% confidence intervals (CIs). Multivariate models were built using a stepwise backward selection process and included only adjusted ORs for significant parameters. The cut-off for the retention of a variable in the model was set at *p* < 0.05. The area under the receiver-operating characteristic (ROC) curve was calculated to examine the overall ability of EAT and established models to predict CAD in T2DM patients. The optimal cut-off values along with corresponding sensitivity, specificity, and predictive values were computed by the Youden index. The DeLong’s test was conducted to compare the performance between models in a pairwise manner [34].

In order to investigate whether EAT could explain the association between circulating miRNA and the presence of CAD in T2DM, causal mediation analysis was performed. To affirm the requirements for mediation analysis specified by Baron and Kenny, a linear regression model for continuous variables (path a) and a logistic regression model for binary outcome (paths b and c) were applied [35]. Then, the R package ‘mediation’ version 4.5.1. was adopted to estimate the total effect of exposure on the outcome decomposed into the average causal mediation effect (ACME), representing the indirect effect through the attributed mediator, and the average direct effect (ADE), according to the algorithms developed by Imai et al. [36, 37]. To obtain 95% CIs, a non-parametric bootstrapping approach (1,000 resamples) with the percentile method was employed. The proportion mediated, representing the proportion of the total effect explained by the mediator, was calculated as the ratio of the ACME to the total effect. Sensitivity analysis for sequential ignorability was performed to test the robustness of the mediator effect to the violations of the assumption, that is, the possible existence of unobserved confounders [36, 37].

All *p*-values were two-tailed, and statistical significance was defined as *p* < 0.05. STATISTICA 13.3.1. (TIBCO Software Inc., Palo Alto, CA, USA), PQStat 1.8.6 (PQStat Software, Poznan, Poland), R version 4.5.1. (R Foundation for Statistical Computing, Vienna, Austria), and GraphPad Prism 10.2.0 (GraphPad Software, San Diego, CA, USA) were used for statistical analysis and image plotting.

## Results

### Baseline characteristics of study and control groups

A total of 94 participants were enrolled in the study, including 30 each in the T2DM-CAD and T2DM groups, 16 CAD patients, and 18 healthy controls. Table 1 shows detailed demographic and clinical characteristics, which have been partially reported in our previous paper [18]. All study participants were matched for age, sex, and BMI. Additionally, bioelectrical impedance analysis revealed no significant intergroup differences in VFR and other body composition parameters (FM, FFM, TBW). Approximately half of the participants in each group fell within the healthy visceral fat range (1–12). The median duration of diabetes was 12 years (IQR 8–16), with a slightly longer duration in T2DM patients with coexisting CAD compared to T2DM individuals, but without statistical significance (*p* = 0.498).

The study groups were well-balanced for conventional cardiovascular risk factors, encompassing blood pressure, uric acid and fibrinogen levels, lipid and renal profiles, the prevalence of hypertension and dyslipidemia. Only HDL-C and homocysteine concentrations varied significantly among the four groups (*p* = 0.002 and *p* = 0.001, respectively). As expected, participants in both diabetes groups had markedly elevated HbA1c levels compared to CAD patients and healthy controls (all *p* < 0.001).

The results of the complete blood count analysis indicated that patients with T2DM-CAD exhibited higher values of NLR and neutrophil counts than controls (*p* = 0.001 and *p* = 0.004, respectively). Importantly, we noted significant differences in NLR and MLR values between T2DM patients with and without CAD (both *p* = 0.017). Other inflammatory parameters, including lymphocyte, monocyte, and platelet counts, PLR value, CRP and IL-6 concentrations appeared comparable across all groups (all *p* > 0.05).

The history of medication use, including antihypertensive agents (angiotensin-converting enzyme inhibitors, angiotensin II receptor blockers, calcium channel blockers, β-blockers), statins, and aspirin was similar in patients with T2DM and/or CAD. There was no significant difference in diabetes treatment regimens between T2DM patients with and without CAD, with similar rates observed for combinations of metformin with either a glucose co-transporter type 2 inhibitor (SGLT-2i) or a glucagon-like peptide-1 receptor agonist (GLP-1RA) (*p* = 0.754).

**Table 1:** Baseline characteristics of the studied and control groups

Variable	T2DM-CAD (*n* = 30)	T2DM (*n* = 30)	CAD (*n* = 16)	Controls (*n* = 18)	*p*-Value
Age [years]	58.27 ± 4.31	57.03 ± 4.46	58.19 ± 2.88	55.61 ± 4.13	0.146^a^
Male, *n* (%)	12 (40.00)	10 (33.33)	9 (56.25)	8 (44.44)	0.503^b^
Duration of T2DM [years]	12.50 ± 5.40	11.57 ± 5.20	–	–	0.498^c^
BMI [kg/m^2^]	29.58 ± 4.08	31.09 ± 2.94	28.56 ± 3.00	29.67 ± 4.22	0.130^a^
WC [cm]	105.10 ± 13.08	110.37 ± 11.82	102.38 ± 8.91	100.11 ± 11.46	0.022^a*^
HC [cm]	106.50 (100.00–114.00)^3*^	113.75 (108.00–121.00)	105.50 (102.50–114.25)	108.50 (100.00–113.00)	0.025^d*^
WHR	0.98 ± 0.09	0.97 ± 0.09	0.95 ± 0.08	0.93 ± 0.10	0.282^a^
FM [kg]	30.00 (24.50–33.60)	33.40 (30.20–35.70)	27.45 (24.25–30.50)	28.25 (24.10–32.50)	0.088^d^
PBF [%]	35.05 (30.50–40.80)	38.75 (34.00–40.80)	33.40 (28.45–37.55)	33.10 (30.90–38.00)	0.087^d^
FFM [%]	64.95 (59.25–69.45)	61.29 (59.24–66.05)	65.87 (61.61–70.09)	66.90 (62.04–69.11)	0.156^d^
TBW [%]	45.95 (42.40–49.20)	40.60 (37.90–47.60)	48.40 (42.10–49.80)	46.55 (42.80–49.80)	0.053^d^
Visceral fat rating	12.53 ± 3.97	13.07 ± 4.09	12.00 ± 3.20	10.72 ± 4.20	0.247^a^
SBP [mmHg]	29.30 ± 7.70	129.83 ± 6.30	129.69 ± 9.71	126.83 ± 6.06	0.552^a^
DBP [mmHg]	76.73 ± 7.26	79.77 ± 5.45	77.19 ± 6.24	81.22 ± 5.16	0.055^a^
HbA1c [%]	7.47 ± 0.78^1,2***^	7.42 ± 1.02^1,2***^	5.31 ± 0.25	5.24 ± 0.25	< 0.001^a***^
TC [mg/dL]	149.47 ± 38.06	161.70 ± 34.68	156.00 ± 33.06	175.17 ± 14.29	0.072^a^
TG [mg/dL]	137.17 ± 47.91	140.77 ± 45.85	123.00 ± 43.92	113.11 ± 38.28	0.156^a^
LDL-C [mg/dL]	78.40 ± 31.27	91.98 ± 30.79	84.90 ± 27.00	97.60 ± 13.95	0.099^a^
HDL-C [mg/dL]	43.63 ± 9.67^1*^	41.57 ± 11.10^1**^	46.50 ± 13.28	54.94 ± 14.16	0.002^a**^
Creatinine [mg/dL]	0.80 ± 0.12	0.73 ± 0.14	0.83 ± 0.09	0.80 ± 0.14	0.054^a^
eGFR [mL/min/1.73 m^2^]	86.70 (75.00–90.00)	90.00 (78.10–90.00)	88.17 (78.63–90.00)	90.00 (78.17–90.00)	0.550^d^
Uric acid [mg/dL]	5.28 ± 1.41	5.80 ± 1.36	5.51 ± 1.39	5.06 ± 1.04	0.252^a^
Homocysteine [µmol/L]	20.56 ± 4.83^1,3**^	16.53 ± 5.42	18.07 ± 3.25	15.32 ± 3.71	0.001^a***^
Fibrinogen [mg/dL]	3.10 (2.70–3.60)	3.10 (2.90–3.70)	3.20 (2.70–4.00)	3.05 (2.60–3.40)	0.530^d^
WBC [10^9^/L]	6.72 ± 1.47	6.64 ± 1.20	6.91 ± 1.48	5.68 ± 1.27	0.032^a*^
NEU [10^9^/L]	4.08 ± 1.05^1**^	3.79 ± 0.96^1*^	4.11 ± 0.99^1*^	2.95 ± 0.71	0.001^a**^
LYM [10^9^/L]	1.71 (1.50–2.16)	2.09 (1.80–2.38)	2.06 (1.54–2.36)	1.96 (1.51–2.22)	0.089^d^
NLR	2.32 ± 0.74^1**,3*^	1.86 ± 0.55	2.13 ± 0.49^1*^	1.51 ± 0.38	< 0.001^a***^
MONO [10^9^/L]	0.40 (0.33–0.49)	0.38 (0.31–0.43)	0.47 (0.39–0.49)	0.36 (0.31–0.45)	0.058^d^
MLR	0.22 (0.19–0.28)^3*^	0.18 (0.15–0.20)	0.22 (0.18–0.31)	0.18 (0.16–0.22)	0.004^d**^
PLT [10^9^/L]	224.47 ± 42.58	236.33 ± 60.42	250.06 ± 59.94	244.00 ± 52.03	0.414^a^
PLR	122.88 (107.29–138.73)	109.88 (97.03–128.57)	128.13 (91.70–172.32)	117.75 (100.51–152.04)	0.520^d^
hs-CRP [mg/L]	1.37 (0.89–2.22)	1.29 (0.50–6.24)	1.31 (0.50–2.04)	0.75 (0.50–1.04)	0.285^d^
IL-6 [pg/mL]	2.70 (2.70–3.10)	2.70 (2.70–2.70)	2.70 (2.70–2.80)	2.70 (2.70–2.70)	0.635^d^
Hypertension, *n* (%)	30 (100.00)	29 (96.67)	16 (100.00)	–	0.460^b^
Dyslipidemia, *n* (%)	28 (93.33)	27 (90.00)	15 (93.75)	–	0.859^b^

Values are presented as number (%) and mean ± SD or median (IQR) based on the data distribution

T2DM-CAD, type 2 diabetes mellitus with coronary artery disease; T2DM, type 2 diabetes mellitus; CAD, coronary artery disease; BMI, body mass index; WC, waist circumference; HC, hip circumference; WHR, waist-to-hip ratio; FM, fat mass; PBF, percentage body fat; FFM, fat-free mass; TBW, total body water; SBP, systolic blood pressure; DBP, diastolic blood pressure; HbA1c, glycated hemoglobin A1c; TC, total cholesterol; TG, triglycerides; LDL-C, low-density lipoprotein cholesterol; HDL-C, high-density lipoprotein cholesterol; eGFR, estimated glomerular filtration rate; WBC, white blood cell; NEU, neutrophil; LYM, lymphocyte; NLR, neutrophil-to-lymphocyte ratio; MONO, monocyte; MLR, monocyte-to-lymphocyte ratio; PLT, platelet; PLR, platelet-to-lymphocyte ratio; hs-CRP, high-sensitivity C-reactive protein; IL-6, interleukin 6; SD, standard deviation; IQR, interquartile range

^a^ One-way ANOVA with Tukey’s post hoc test for unequal *n*

^b^ Chi-Squared test

^c^ Student’s *t*-test

^d^ Kruskal-Wallis test followed by Dunn’s test

^1^ Significantly different from controls; ^2^ Significantly different from CAD; ^3^ Significantly different from T2DM. *p*-value < 0.05 was statistically significant. ^*^p < 0.05; ^**^p < 0.01; ^***^p < 0.001

### Echocardiographic parameters and epicardial adipose tissue thickness

There were no differences in the LVESD, LVEDD, and LVEDV between the four patient subgroups (all *p* > 0.05). The LVEF was within the normal range (LVEF ≥ 52% for men and ≥ 54% for women) in all study participants. Compared with the healthy controls, patients with T2DM-CAD exhibited significantly lower LVEF (*p* = 0.007). Likewise, the FS (*p* = 0.017) and LVESV (*p* = 0.046) showed statistically significant differences between the T2DM-CAD and control groups, while similarities were observed in the remaining intergroup pairwise comparisons. Echocardiographic findings in the study subjects are depicted in Table 2.

**Table 2:** Standard echocardiographic parameters and EAT measurements in the study population

Variable	T2DM-CAD (*n* = 30)	T2DM (*n* = 30)	CAD (*n* = 16)	Controls (*n* = 18)	*p*-Value
LVESD [mm]	31.86 ± 4.20	30.57 ± 4.85	30.34 ± 3.45	29.14 ± 4.15	0.205^a^
LVEDD [mm]	49.45 ± 4.84	49.46 ± 4.94	49.16 ± 3.87	49.17 ± 4.63	0.994^a^
FS [%]	35.62 ± 5.06^1*^	38.45 ± 5.52	38.30 ± 4.53	40.81 ± 5.12	0.010^a*^
LVESV [mL]	37.92 (33.31–51.22)^1*^	35.72 (29.03–47.10)	34.72 (28.65–45.93)	29.03 (26.28–34.15)	0.062^b^
LVEDV [mL]	116.75 ± 26.47	116.89 ± 26.73	114.60 ± 20.31	115.08 ± 24.67	0.987^a^
EF [%]	64.09 ± 6.15^1**^	68.15 ± 6.82	68.13 ± 5.62	71.11 ± 6.23	0.003^a**^
EAT [mm]	7.78 (6.97–9.41)^1,2****^	5.46 (5.05–6.76)	6.91 (5.41–8.05)^1**^	4.89 (4.16–5.31)	< 0.0001^b****^
EAT-PLAX [mm]	7.99 (7.21–10.20)^1,2****^	5.67 (5.21–6.88)	6.88 (5.61–8.23)^1**^	5.03 (4.51–5.36)	< 0.0001^b****^
EAT-PSAX [mm]	7.85 (6.50–9.61)^1,2****^	5.43 (4.59–6.45)	6.77 (5.05–7.87)^1**^	4.75 (4.33–5.26)	< 0.0001^b****^

Data are presented as mean ± SD or median (IQR)

T2DM-CAD, type 2 diabetes mellitus with coronary artery disease; T2DM, type 2 diabetes mellitus; CAD, coronary artery disease; LVESD, left ventricular end-systolic diameter; LVEDD, left ventricular end-diastolic diameter; FS, fractional shortening; LVESV, left ventricular end-systolic volume; LVEDV, left ventricular end-diastolic volume; EF, ejection fraction; EAT, epicardial adipose tissue; PLAX, parasternal long axis; PSAX, parasternal short axis; SD, standard deviation; IQR, interquartile range

^a^ One-way ANOVA with Tukey’s post hoc test for unequal *n*

^b^ Kruskal-Wallis test followed by Dunn’s test

^1^ Significantly different from controls; ^2^ Significantly different from T2DM. *p*-value < 0.05 was statistically significant. ^*^p < 0.05; ^**^p < 0.01; ^***^p < 0.001; ^****^p < 0.0001

Bland-Altman analysis demonstrated that the agreement between long- and short-axis epicardial fat thickness measurements was good with a bias of 0.45 (95% CI, −0.79–1.69) (Additional file 1: Figure S1). The concordance correlation coefficient of EAT measurements in the two projections was 0.96 (95% CI, 0.94–0.97) with the precision reaching 0.97 and the accuracy of 0.98. Therefore, the average of EAT measurements from both axes was considered for subsequent analysis. The intra- and interobserver correlation coefficients of EAT measurements in the parasternal long- and short-axis views were 0.98 and 0.95, respectively, indicating good reproducibility and reliability (Additional file 1: Table S1).

**Fig. 2:**
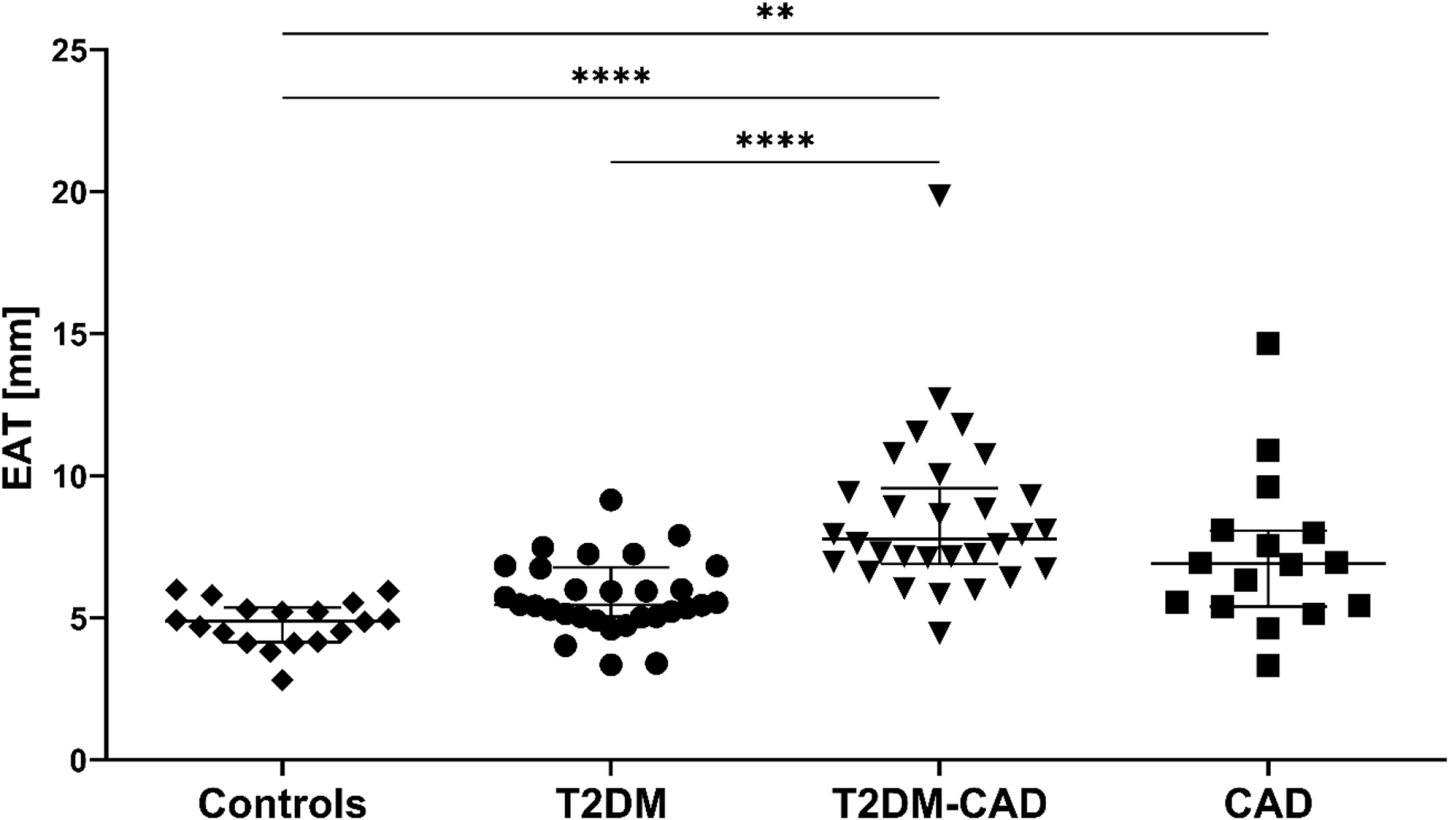
Scatter plot illustrating EAT thickness in patients with T2DM-CAD, T2DM, CAD, and healthy controls. Data are presented as median (IQR). *p*-value < 0.05 was statistically significant. ^**^p < 0.01; ^****^p < 0.0001. The analysis was performed using Kruskal-Wallis test followed by Dunn’s test. T2DM-CAD, type 2 diabetes mellitus with coronary artery disease; T2DM, type 2 diabetes mellitus; CAD, coronary artery disease; EAT, epicardial adipose tissue; IQR, interquartile range

Echocardiographic examination revealed an increasing stepwise trend in EAT thickness from the control group, through T2DM, CAD to the T2DM-CAD group (Table 2; Fig. 2). The median EAT thickness was significantly higher in T2DM-CAD patients compared to T2DM subjects and controls (both *p* < 0.0001). Additionally, we observed a significant difference in epicardial fat depots between participants diagnosed with CAD and healthy controls (*p* = 0.003).

In the overall study population, the distribution of EAT ranged from 2.82 mm to 19.85 mm, with no significant difference between men and women (*p* = 0.593) (Additional file 1: Figure S2A). EAT thickness was also not statistically different between obese and non-obese participants in the four analyzed groups (all *p* > 0.05) (Additional file 1: Figure S3A-D). Moreover, EAT thickness turned out to be similar regardless of the antidiabetic treatment used (metformin + SGLT-2i vs. metformin + GLP1-RA, *p* = 0.158) (Additional file 1: Figure S2B).

### Differences in miRNA expression levels according to epicardial adipose tissue thickness

The expression of hsa-miR-4505 and hsa-miR-4743-5p was significantly up-regulated, while hsa-miR-4750-3p was down-regulated in T2DM-CAD patients compared to T2DM and control subjects, as previously discovered [18]. Table S2 presents the detailed findings on plasma miRNA expression analysis by RT-qPCR [18].

**Fig. 3:**
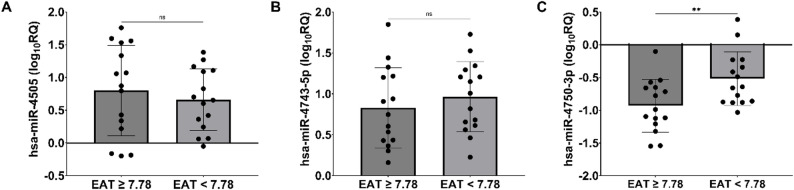
Comparison of hsa-miR-4505, hsa-miR-4743-5p, hsa-miR-4750-3p expression in T2DM-CAD patients according to the median EAT thickness. **A** hsa-miR-4505; **B** hsa-miR-4743-5p; **C** hsa-miR-4750-3p. Data are presented as mean ± SD. *p*-value < 0.05 was statistically significant. ^**^p < 0.01. The analysis was performed using Student’s *t*-test. EAT, epicardial adipose tissue; log_10_RQ, logarithmically transformed relative expression; SD, standard deviation; ns, no significance

After categorizing T2DM-CAD patients according to the median EAT thickness (7.78 mm), hsa-miR-4750-3p expression level was found to be significantly lower in the group with the greater EAT thickness measured by echocardiography (*p* = 0.010). No significant differences were observed for hsa-miR-4505 and hsa-miR-4743-5p (*p* > 0.05) with respect to EAT thickness, as shown in Fig. 3.

### Correlation of epicardial adipose tissue thickness with miRNAs and clinical data

A Spearman’s rank-order correlation was conducted to determine the association between EAT thickness and circulating miRNAs and conventional CAD risk factors. Considering the combined group of T2DM patients, both with and without CAD, EAT thickness positively correlated with the expression of hsa-miR-4505 and hsa-miR-4743-5p (*ρ* = 0.380, *p* = 0.003 and *ρ* = 0.325, *p* = 0.011, respectively), while it was inversely associated with hsa-miR-4750-3p (*ρ* = −0.556, *p* < 0.001). EAT thickness also significantly linked to VFR (*ρ* = 0.259, *p* = 0.046) and indicators of subclinical inflammation, such as NLR and MLR (*ρ* = 0.373 and *ρ* = 0.380, respectively, both *p* = 0.003). Additionally, NLR was proved to be significantly correlated with the expression of hsa-miR-4743-5p and hsa-miR-4750-3p (*ρ* = 0.335, *p* = 0.009 and *ρ* = −0.320, *p* = 0.013), while MLR was only connected with hsa-miR-4750-3p (*ρ* = −0.299, *p* = 0.020).

**Fig. 4:**
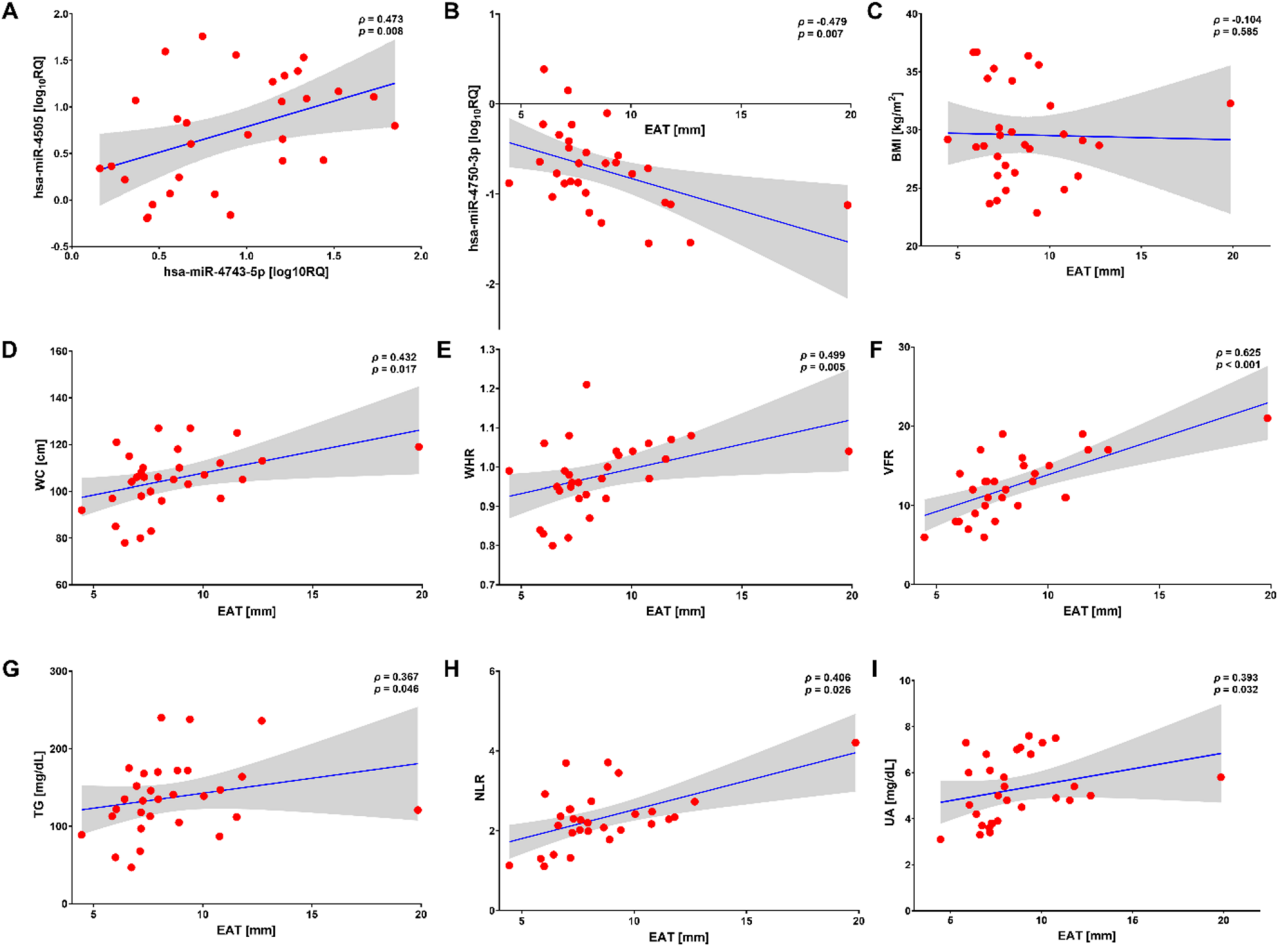
Scatter plots demonstrating correlations between EAT thickness, miRNAs, and clinical data in patients with T2DM-CAD. **A** The statistically significant relationship between hsa-miR-4505 and hsa-miR-4743-5p, and **B** the inverse correlation between hsa-miR-4750-3p and EAT thickness were detected. **C** There was no significant correlation between EAT thickness and BMI. **D–I** The statistically significant positive correlations were observed between EAT thickness and WC, WHR, VFR, TG, NLR, UA. The blue line represents the linear regression slope with 95% CI (grey area). *p*-value < 0.05 was statistically significant. T2DM-CAD, type 2 diabetes mellitus with coronary artery disease; log_10_RQ, logarithmically transformed relative expression; EAT, epicardial adipose tissue; BMI, body mass index; WC, waist circumference; WHR, waist-to-hip ratio; VFR, visceral fat rating; TG, triglycerides; NLR, neutrophil-to-lymphocyte ratio; UA, uric acid; CI, confidence interval

A similar observation was found in the T2DM-CAD group, in which there was a moderate positive correlation between the expression levels of hsa-miR-4505 and hsa-miR-4743-5p (Fig. 4A). In this group, EAT thickness significantly positively correlated with anthropometric parameters (WC, WHR, VFR), TG and uric acid concentrations, NLR value, whereas it was inversely linked to hsa-miR-4750-3p expression (Fig. 4B–I). All miRNAs under study were significantly associated with LDL-C concentrations (*p* < 0.05). The bivariate analysis revealed that hsa-miR-4750-3p expression decreased with increasing TG levels (*ρ* = −0.420, *p* = 0.021). Moreover, we detected a positive correlation of TC concentrations with hsa-miR-4505 and hsa-miR-4743-5p (*ρ* = 0.416, *p* = 0.022 and *ρ* = 0.398, *p* = 0.029, respectively), and an inverse one of marginal statistical significance with hsa-miR-4750-3p (*ρ* = −0.343, *p* = 0.063), supporting their interrelation with lipid metabolism (Additional file 1: Figure S4A).

In addition, EAT thickness showed a significant negative association with hsa-miR-4750-3p expression in both CAD patients and healthy individuals. With regard to visceral obesity indices, epicardial fat thickness was positively associated with VFR and WC, WHR, and VFR in the CAD group and the control group, respectively (Additional file 1: Figure S4B-C).

### Identification of independent determinants of epicardial adipose tissue thickness

As presented in Table 3, all analyzed miRNAs were significantly linked to EAT thickness in univariate linear regression (*p* < 0.05). To further identify independent predictors of EAT thickness, we developed three sequential multivariate linear regression models. The unadjusted model (model 1) revealed a negative association between the expression of hsa-miR-4750-3p and EAT thickness (*β* = −0.481, *p* < 0.001), which remained significant (*β* = −0.454, *p* < 0.001) even after adjusting for relevant confounders (model 2), including age, sex, duration of diabetes, and anthropometric parameters. In the fully adjusted model (model 3), after accounting for additional cardiometabolic factors and inflammatory indices, hsa-miR-4750-3p (*β* = − 0.445, *p* = 0.003) along with NLR (*β* = 0.366, *p* = 0.037) emerged as independent determinants of EAT thickness.

**Table 3:** Univariate and multivariate linear regression analysis of the association between EAT thickness and miRNA expression

Variable	EAT thickness								
	Univariate			Model 1		Model 2		Model 3	
	*β* (95%CI)		*p*-Value	*β* (95%CI)	*p*-Value	*β* (95%CI)	*p*-Value	*β* (95%CI)	*p*-Value
hsa-miR-4505	0.448 (0.213 to 0.683)		< 0.001^*^	0.266 (−0.006 to 0.539)	0.055	0.253 (−0.022 to 0.527)	0.070	0.172 (−0.135 to 0.480)	0.264
hsa-miR-4743-5p	0.297 (0.046 to 0.548)		0.021^*^	−0.019 (−0.283 to 0.244)	0.884	−0.023 (−0.305 to 0.260)	0.873	−0.033 (−0.349 to 0.282)	0.833
hsa-miR-4750-3p	−0.582 (−0.796 to −0.368)		< 0.001^*^	−0.481 (−0.710 to −0.252)	< 0.001^*^	−0.454 (−0.686 to −0.223)	< 0.001^*^	−0.445 (−0.727 to −0.163)	0.003^*^

Model 1: unadjusted model; Model 2: adjusted for age, sex, duration of T2DM, BMI, WHR, VFR; Model 3: adjusted for the same variables as model 2, with additional covariates, including HbA1c, TC, TG, HDL-C, WBC, NLR, MLR, PLR, hs-CRP, IL-6, uric acid

EAT, epicardial adipose tissue; CI, confidence interval; T2DM, type 2 diabetes mellitus; BMI, body mass index; WHR, waist-to-hip ratio; VFR, visceral fat rating; HbA1c, glycated hemoglobin A1c; TC, total cholesterol; TG, triglycerides; HDL-C, high-density lipoprotein cholesterol; WBC, white blood cell; NLR, neutrophil-to-lymphocyte ratio; MLR, monocyte-to-lymphocyte ratio; PLR, platelet-to-lymphocyte ratio; hs-CRP, high-sensitivity C-reactive protein; IL-6, interleukin 6

^*^p-value < 0.05 was statistically significant. The relative expression of miRNA and EAT thickness were logarithmically transformed (log_10_ and natural log, respectively). Candidate variables for multivariate models were selected based on clinical grounds, a *p*-value < 0.10 in univariate linear regression, and the absence of collinearity

### Factors associated with higher risk of coronary artery disease in type 2 diabetes mellitus

To investigate which demographic, laboratory, echocardiographic, and molecular parameters were most associated with CAD occurrence in T2DM, the logistic regression modeling was performed.

The following eight variables showed a significant relationship with CAD in the univariate analysis: hsa-miR-4505, hsa-miR-4743-5p, and hsa-miR-4750-3p expression levels, NLR and MLR values, homocysteine and hs-CRP concentrations, and EAT thickness (Table 4). In the multivariate analysis, increased expression of hsa-miR-4505 (OR, 6.555; 95% CI, 1.004–42.804; *p* = 0.049) and hsa-miR-4743-5p (OR, 3.383; 95% CI, 1.075–10.641; *p* = 0.037), decreased expression of hsa-miR-4750-3p (OR, 0.178; 95% CI, 0.039–0.808; *p* = 0.025), and greater EAT thickness (OR, 5.029; 95% CI, 1.423–17.771; *p* = 0.012) were independently associated with higher CAD risk in patients with T2DM.

**Table 4:** Univariate and multivariate logistic regression analysis identifying predictors of CAD in T2DM

Variable	Univariate		Multivariate	
	OR (95% CI)	*p*-Value	OR (95% CI)	*p*-Value
Age	1.068 (0.948–1.204)	0.278		
Male	1.333 (0.465–3.823)	0.593		
Duration of T2DM	1.035 (0.939–1.141)	0.491		
hsa-miR-4505	2.743 (1.662–4.528)	< 0.001*	6.555 (1.004–42.804)	0.049*
hsa-miR-4743-5p	2.868 (1.661–4.954)	< 0.001*	3.383 (1.075–10.641)	0.037*
hsa-miR-4750-3p	0.374 (0.225–0.623)	< 0.001*	0.178 (0.039–0.808)	0.025*
BMI	0.885 (0.762–1.027)	0.108		
WC	0.966 (0.925–1.008)	0.111		
WHR	4.081 (0.012–1442.381)	0.639		
VFR	0.967 (0.851–1.099)	0.604		
HbA1c	1.069 (0.607–1.883)	0.817		
TC	0.991 (0.976–1.005)	0.197		
TG	0.998 (0.987–1.009)	0.763		
HDL-C	1.020 (0.970–1.072)	0.439		
LDL-C	0.986 (0.969–1.003)	0.099		
Homocysteine	1.168 (1.043–1.308)	0.007*		
Fibrinogen	0.565 (0.243–1.311)	0.184		
Uric acid	0.756 (0.516–1.106)	0.150		
WBC	1.050 (0.715–1.541)	0.805		
NLR	3.174 (1.249–8.066)	0.015*		
MLR	15358.944 (2.455–96095154.333)	0.031*		
PLR	1.010 (0.994–1.027)	0.210		
hs-CRP	0.785 (0.630–0.977)	0.030*		
IL-6	1.221 (0.855–1.744)	0.272		
EAT	2.875 (1.636–5.052)	< 0.001*	5.029 (1.423–17.771)	0.012*

T2DM, type 2 diabetes mellitus; CAD, coronary artery disease; OR, odds ratio; CI, confidence interval; BMI, body mass index; WC, waist circumference; WHR, waist-to-hip ratio; VFR, visceral fat rating; HbA1c, glycated hemoglobin A1c; TC, total cholesterol; HDL-C, high-density lipoprotein cholesterol; TG, triglycerides; LDL-C, low-density lipoprotein cholesterol; WBC, white blood cell; NLR, neutrophil-to-lymphocyte ratio; MLR, monocyte-to-lymphocyte ratio; PLR, platelet-to-lymphocyte ratio; hs-CRP, high-sensitivity C-reactive protein; IL-6, interleukin 6; EAT, epicardial adipose tissue

^*^p-value < 0.05 was statistically significant. The relative expression of miRNAs was logarithmically transformed (log_2_)

### Assessment of epicardial adipose tissue for its diagnostic potential

ROC curve analysis was performed to explore the potential significance of echocardiographically measured EAT thickness in predicting CAD in T2DM patients. EAT thickness managed to differentiate T2DM-CAD patients from those with T2DM with an area under the curve (AUC) of 0.869 (95% CI, 0.778–0.961; *p* < 0.001). The optimal cut-off point was set at 6.04 with a sensitivity of 90.00% and specificity of 73.33%, indicating good diagnostic performance for CAD detection (Additional file 1: Figure S5). Additionally, EAT thickness demonstrated the ability not only to detect CAD in patients with T2DM, but also to discriminate patients with CAD alone from healthy individuals (AUC = 0.840; 95% CI, 0.696–0.984; *p* < 0.001). The highest diagnostic performance of EAT was observed in differentiating T2DM-CAD patients from those in the control group (AUC = 0.971; 95% CI, 0.923–1.000; *p* < 0.001) (Additional file 1: Figure S5).

### Combining epicardial adipose tissue with miRNAs and clinical data for coronary artery disease detection in type 2 diabetes mellitus

To explore, whether adding clinical and/or molecular traits to epicardial fat thickness enhances the ability to distinguish between T2DM patients with and without CAD, logistic regression models with a stepwise backward selection process were developed. Considering the significant CAD risk factors identified in univariate logistic regression, we constructed five models. Model 1 was based on EAT thickness, model 2 included only clinical variables (homocysteine, hs-CRP, NLR), and model 3 integrated both echocardiographic and clinical factors (EAT, homocysteine). Model 4 linked molecular traits (hsa-miR-4505, hsa-miR-4743-5p, hsa-miR-4750-3p) to EAT, while model 5 extended it by incorporating clinical parameters.

**Fig. 5:**
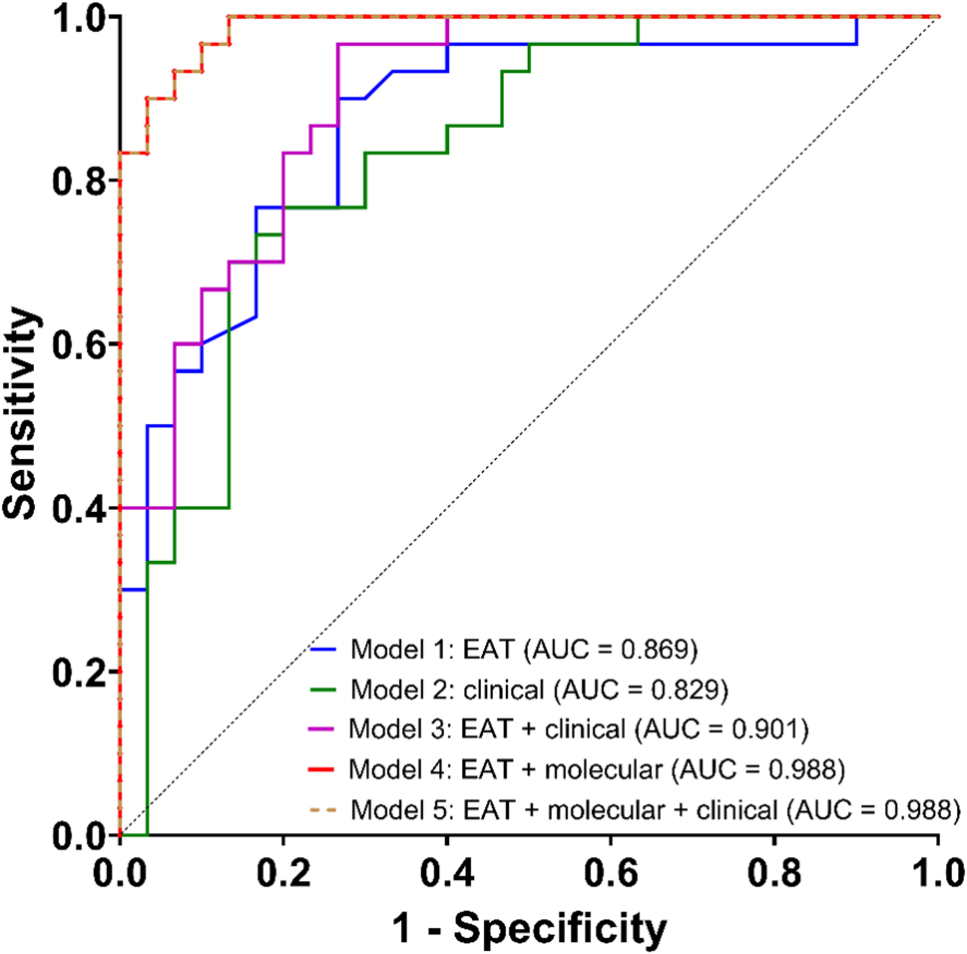
ROC curves of the constructed models for differentiating patients with T2DM-CAD from those with T2DM. Model 1: EAT; Model 2: homocysteine, NLR, hs-CRP; Model 3: EAT, homocysteine; Model 4 and 5: EAT, hsa-miR-4505, hsa-miR-4743-5p, hsa-miR-4750-3p. ROC, receiver operating characteristic; AUC, area under the curve; T2DM-CAD, type 2 diabetes mellitus with coronary artery disease; T2DM, type 2 diabetes mellitus; EAT, epicardial adipose tissue; NLR, neutrophil-to-lymphocyte ratio; hs-CRP, high-sensitivity C-reactive protein

Subsequently, ROC curve analysis was conducted to assess the diagnostic potential of the constructed models. As shown in Fig. 5, the combination of EAT thickness and clinical parameters (model 3) provided better classification capacity compared to each component separately (models 1 and 2), albeit without statistical significance (*p* > 0.05). The integration of EAT thickness with miRNA expression (model 4) substantially enhanced predictive power, resulting in a higher AUC of 0.988 compared to models 1–3 (*p* = 0.013, *p* = 0.004, and *p* = 0.026, respectively), as well as miRNA-based model (AUC = 0.959) [18], but this improvement did not reach statistical significance (*p* = 0.240). It is worth noting that combining EAT with molecular and clinical parameters (model 5) yielded the same components as model 4, without improving the model’s identification efficacy (AUC = 0.988). Hence, model 4 achieved the most encouraging results for discriminating between T2DM-CAD and T2DM patients, with 100% sensitivity and 86.67% specificity. Additionally, model 4 exhibited the lowest standard error (SE = 0.009) and the most favourable corrected Akaike information criterion (AICc = 25.30), indicating good model fit and discrimination ability. The basic parameters and common quality measures of the established models are summarized in Table S3, while the comparison of ROC curves of the models are presented in Table S4.

### Mediating effect of epicardial adipose tissue on the association between hsa-miR-4750-3p and coronary artery disease in type 2 diabetes mellitus

Based on the logistic regression results, it was noted that all three circulating miRNAs were independently associated with the presence of CAD in T2DM, with reduced expression of hsa-miR-4750-3p increasing the risk of CAD with a log odds coefficient (β) of −3.265 (95% CI, −4.957 to −1.572; *p* < 0.001, path c). Additionally, only hsa-miR-4750-3p emerged as a significant negative predictor of EAT thickness in linear regression (unstandardized coefficient: −0.385; 95% CI, −0.527 to −0.244; *p* < 0.001, path a), therefore, hsa-miR-4750-3p was set as an independent variable in the subsequent mediation analysis. After including EAT in the model, the link between hsa-miR-4750-3p and the outcome persisted, with EAT emerging as an independent determinant of CAD in T2DM (log odds β: 5.741, 95% CI, 2.006–9.476; *p* = 0.003, path b), supporting its presupposed mediating role.

**Fig. 6:**
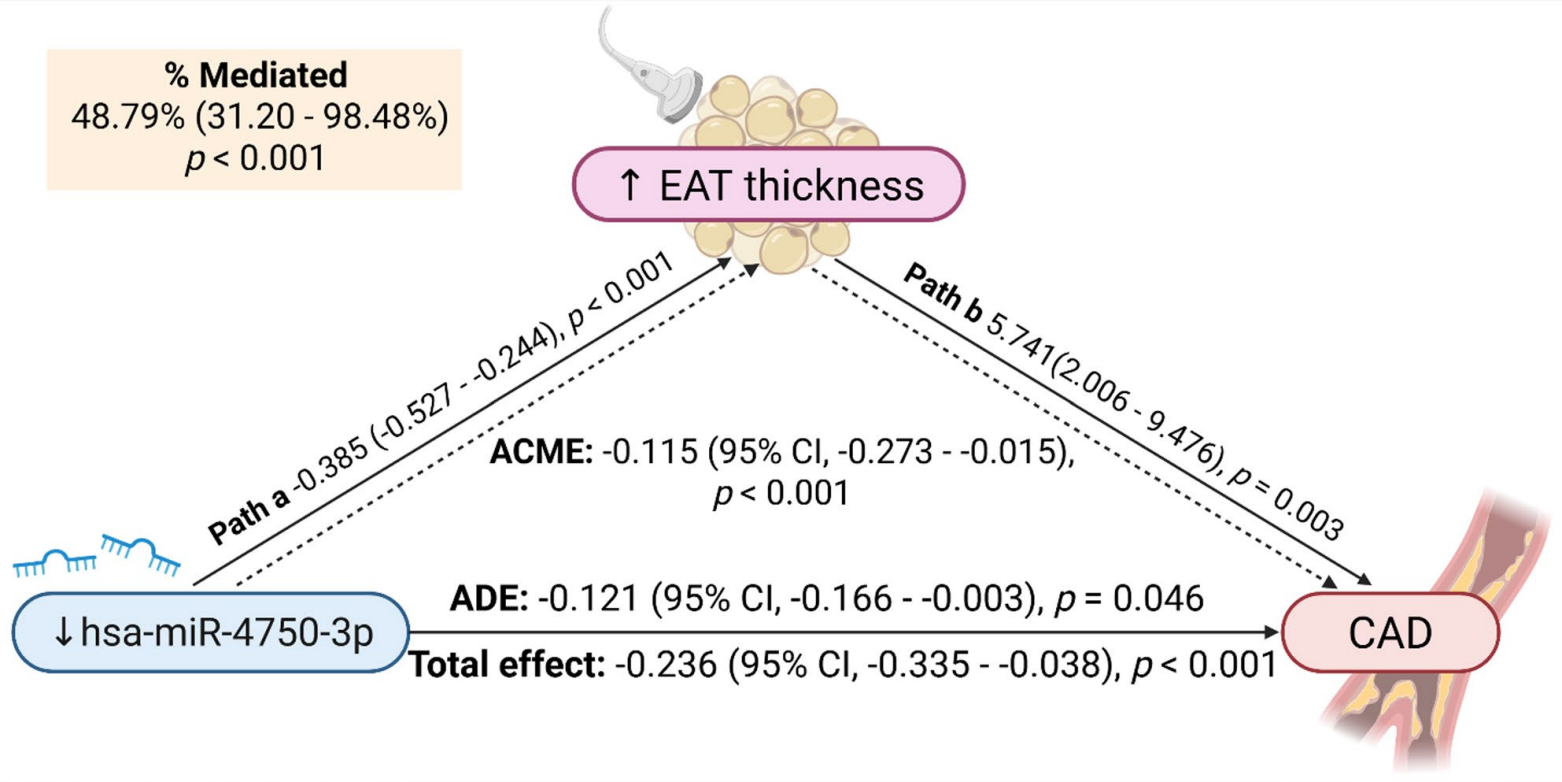
Mediating effect of EAT on the association between hsa-miR-4750-3p and CAD presence in T2DM. The ACME of hsa-miR-4750-3p on CAD in T2DM through EAT was significant, indicating that roughly half of the total effect of miRNA on CAD presence in patients with T2DM was mediated by increased EAT thickness. The relative expression of miRNA and EAT thickness were logarithmically transformed (log_10_ and natural log, respectively). The coefficient for the linear regression model (path a) is given as unstandardized regression coefficient, while that for logistic regression (path b) is reported as log odds coefficient (β). Created with BioRender.com, accessed on 24 September 2025. EAT, epicardial adipose tissue; CAD, coronary artery disease; T2DM, type 2 diabetes mellitus; CI, confidence interval; ACME, average causal mediation effect; ADE, average direct effect

Given that the prerequisite conditions for mediation were met solely for hsa-miR-4750-3p, we exploratorily tested whether greater EAT thickness could explain the association between dysregulated hsa-miR-4750-3p expression and CAD in T2DM. Causal mediation analysis revealed the significant ACME estimated at −0.115 (95% CI, −0.273 to −0.015; *p* < 0.001). It was also found that hsa-miR-4750-3p exerts the direct impact on CAD independent of epicardial fat, reflected by the ADE of −0.121 (95% CI, − 0.166 to − 0.003; *p* = 0.046). As depicted in Fig. 6, the proportion of the total effect mediated by EAT was 48.79% (95% CI, 31.20–98.48%; *p* < 0.001), suggesting that EAT could partially explain the linkage between hsa-miR-4750-3p and the presence of CAD in T2DM.

To evaluate the robustness of our estimates to potential violations of the assumption regarding unmeasured confounding factors, we conducted a sensitivity analysis for sequential ignorability. Although the ACME was statistically significant at *ρ* = 0, the indirect effect would be reduced to zero under a moderate degree of unmeasured mediator-outcome confounding (*ρ* = 0.30), corresponding to a shared residual variance of 0.09 (Additional file 1: Figure S6).

## Discussion

In the present study, we attempted to determine the crosstalk between EAT and three fatty acid metabolism-related miRNAs in the complex process of diabetic atherosclerosis. Our findings demonstrated that EAT thickness was significantly greater in T2DM-CAD individuals compared to T2DM patients and healthy controls. Additionally, a link between lipid indices with both EAT and miRNAs and EAT with NLR was observed, suggesting the involvement of disturbed lipid metabolism and subclinical inflammation in EAT-related CAD. To the best of our knowledge, this study is the first to reveal an association between EAT thickness and the expression of circulating hsa-miR-4750-3p, which, along with hsa-miR-4505 and hsa-miR-4743-5p, were identified as independent predictors of CAD in T2DM. Innovatively, we proposed EAT as a potential mediator between reduced hsa-miR-4750-3p expression and diabetes-related CAD, supporting the concept of the miRNA-adipose tissue axis that may underlie enhanced atherosclerosis susceptibility in T2DM.

Since the discovery of EAT by Iacobellis et al., research on cardiometabolic diseases has shown a step back from BMI-centric obesity, known as an established cardiovascular risk factor, to organ-specific adiposity [6]. EAT constitutes one of the major components of cardiac fat, which covers nearly 80% of the heart surface, predominantly on the free wall of the RV [38, 39]. As an imaging biomarker, epicardial fat can be easily measured by echocardiography, and its thickness varied from a minimum of 1 mm to a maximum of almost 25 mm [6, 31]. Consistent with previous reports, the thickness of EAT in our study population exhibited a wide range from 2.82 mm to 19.85 mm, indicating a physiological and pathological epicardial fat depot in humans. We noted a stepwise increase in EAT thickness across the study groups, with the lowest measurements in controls, through the T2DM and CAD groups, to the most pronounced values in T2DM-CAD individuals. The differences remained statistically significant between T2DM patients with and without CAD, mirroring earlier findings in which higher epicardial fat accumulation was linked to both the presence and severity of CAD [40–42]. The considerable thickening of EAT observed in CAD patients, irrespective of diabetes status, compared to healthy controls suggests that it could aid in identifying atherosclerotic burden not only in individuals with T2DM, but also in the general population [43–46].

Even though cardiac magnetic resonance (CMR) and multidetector computed tomography (MDCT) are the gold standard for the quantification of EAT, echocardiography still remains the preferred method in daily clinical practice, favored for its non-invasiveness, low cost, accessibility, and lack of radiation burden to the patient, but limited by its operator-dependent nature [6, 47]. Nevertheless, similar to the majority of population-based clinical studies, we demonstrated high inter- and intra-observer agreement for EAT thickness measurements by echocardiography, highlighting its good reproducibility and reliability [30, 31, 48–51]. Regardless of how it is measured, EAT has been found to correlate with several anthropometric indices, including WC, WHR, VFR, and, to a lesser extent, overall obesity reflected by BMI, in different clinical populations [29–31, 52–55]. Likewise, in our patients with T2DM-CAD, EAT thickness demonstrated associations with all of these adiposity measures except BMI. Taken together, these insights reinforce the potential of EAT as a novel, precise indicator of true visceral fat content.

EAT, accounting for only 1% of total fat mass, functions for more than a simple fat storage depot, but is also a source of various bioactive molecules such as adipokines, cytokines, chemokines, lipid species, and miRNAs [6, 12, 39]. The abnormal enlargement of epicardial fat in T2DM patients with CAD has been hypothesized to be due to higher lipoprotein lipase activity, being responsible for catabolism of TG from chylomicrons and VLDL, thereby increasing the influx of FFAs into epicardial adipocytes and affecting systemic lipoprotein metabolism [56, 57]. The loss of brown fat-like features of EAT in the diabetic milieu impairs fatty acid buffering for the underlying myocardium, lipid accumulation and β-oxidation, exacerbating insulin resistance and inflammatory response [58–61]. Moreover, it has been found that epicardial adipocytes from CAD patients with T2DM exhibited decreased expression of genes encoding master regulators of oxidative metabolism, including peroxisome proliferator-activated receptor gamma coactivator 1-alpha (PGC1α) [61]. Importantly, PGC1α expression in EAT was positively correlated with the levels of anti-inflammatory and anti-atherosclerotic HDL-C, while it was negatively correlated with circulating TG levels [61]. A decline in β-oxidation of FFAs in EAT may have a dual detrimental effect on CAD occurrence in diabetic patients [61]. Firstly, decreased clearance of circulating TG and lipid-induced inflammation and cardiotoxicity foster atherosclerotic plaque development [61, 62]. Secondly, the concurrent reduction in energy availability for the myocardial cells may aggravate its dysfunction [61, 62]. Our results align with prior reports, as we demonstrated that increased thickness of epicardial fat is concomitant with elevated TG levels, implying a reciprocal interaction between EAT expansion and altered lipid metabolism in T2DM-CAD patients. Although we did not observe a correlation between HDL-C concentration and EAT thickness, T2DM patients with and without CAD had significantly lower HDL-C levels compared to healthy individuals. Similar plasma HDL-C concentrations detected in both T2DM groups can be partially explained by literature data emphasizing that the increased risk of CAD in T2DM may not be related solely to the HDL-C quantity, but also to alterations in the composition of HDL affecting its function [63, 64].

Numerous biomolecular studies have shown that epicardial fat exhibits an intrinsic inflammatory profile, playing a central role in coronary artery endothelial cell dysfunction in T2DM [11, 65, 66]. Leveraging transcriptome and secretome analyses of EAT from T2DM and CAD patients, increased expression of pro-inflammatory adipokines and cytokines, such as IL-6, interleukin-8 (IL-8), tumor necrosis factor α (TNF-α), and monocyte chemoattractant protein-1 (MCP-1) was detected [66–68]. In contrast to earlier reports, serum IL-6 levels were comparable across all groups in our study, suggesting its predominant localization in epicardial adipocytes and adjacent coronary vasculature [67, 69, 70]. It is widely recognized that inflamed EAT is characterized by a dense infiltration of leukocytes, particularly CD8+ T cells, mast cells, and macrophages, with a significant shift toward their pro-inflammatory M1 phenotype [6, 56, 71]. Here, we revealed that patients with T2DM-CAD had higher levels of low-grade inflammation compared to those with T2DM, as evidenced by elevated NLR and MLR values. Intriguingly, it has been shown that increased epicardial adiposity may be associated with a more pronounced inflammatory response and, consequently, with more advanced coronary artery atherosclerosis [6, 67, 72]. Along the same lines, we confirmed that the thicker the EAT layer, the higher the NLR value in T2DM-CAD patients. Similarly, Akbas et al. noted a positive association between EAT thickness and NLR values in diabetic patients [73]. Beyond this, Liu et al. found that diminished EAT density reflecting systemic low-grade inflammation was linked to increased NLR values in individuals with CAD [74]. These observations suggest that not only the presence of inflammatory cell infiltration, but also the relative proportion of specific immune cells may serve as a potential indicator of subclinical inflammation and coronary atherosclerosis.

Given the growing importance of miRNome in the regulation of lipid metabolism and inflammation, its association with EAT in promoting diabetic atherosclerosis has become of particular interest [17, 24, 25]. A recent study has evidenced that the miRNA expression profile was similar in EAT explants, conditioned media and plasma samples from CAD patients, supporting the concept that specific miRNAs identified in the circulation may be secreted by epicardial fat [75]. However, it is still little known about circulating miRNAs in EAT-related atherosclerosis in T2DM. The present study expands on our previous findings, which unveiled hsa-miR-4505, hsa-miR-4743-5p, and hsa-miR-4750-3p to be significantly dysregulated in the plasma of T2DM-CAD patients compared to T2DM subjects [18]. Although we have not investigated the exact source, potential targets, and mechanisms by which miRNAs are transported into circulation, we have shown that when hsa-miR-4505 and hsa-miR-4743-5p are up-regulated, and hsa-miR-4750-3p is down-regulated, EAT thickness consistently increases in the entire diabetic population. Likewise, de Gonzalo-Calvo et al. revealed that the expression of miR-15b-3p, miR-22-3p, miR-148a-3p, and miR-148b-3p in the plasma of cardiac patients was positively associated with EAT volume [75]. Nonetheless, after adjusting for relevant cardiometabolic confounders, the relationship observed in our study retained statistical significance exclusively for hsa-miR-4750-3p, which was identified as an independent determinant of EAT thickness. This suggests that the contribution of hsa-miR-4505 and hsa-miR-4743-5p to CAD in T2DM may arise from different mechanisms beyond epicardial fat dysfunction.

Based on an in-depth correlation analysis, we discovered that circulating hsa-miR-4750-3p is inversely associated with TG in patients with T2DM-CAD. What is more, each of the three DE-miRNAs shared a common association with LDL-C, which is known for its ability to promote inflammation, oxidative stress, and ultimately atherosclerosis [76]. The observed relationship between miRNAs and lipid profile is further supported by prior functional enrichment analyses of the DE-miRNA target genes, highlighting their key role in regulating fatty acid metabolism, particularly fatty acid oxidation and degradation [18]. It strengthens the potential relevance of specific miRNAs in triggering epicardial fat dysfunction, systemic lipid dysregulation, and increased cardiovascular risk in T2DM. Concurrently, the correlations between hsa-miR-4743-5p and hsa-miR-4750-3p with NLR and hsa-miR-4750-3p with MLR in the combined diabetic group were detected, reflecting their multi-target nature and an additional inflammatory dimension. These findings are consistent with existing scientific evidence pointing to the involvement of EAT-related miRNAs in modulating inflammatory processes [77–80].

Although there are multiple pathways through which EAT could exert its detrimental effect on coronary atherosclerosis, circulating hsa-miR-4750-3p may serve as a potential driver within this association. The mediation analysis in this study revealed that approximately half of the effect of hsa-miR-4750-3p on CAD in patients with T2DM can be attributed to EAT. This aligns with the results of a previous experimental study, which identified perivascular adipose tissue as a mediator of miRNA impact on atherosclerosis progression [81]. Similarly, Burke et al. showed that the extracellular vesicle–mediated transfer of muscle-specific miR-1 to adipose tissue targets genes and enhances lipolytic pathways [15]. Our findings, albeit exploratory in nature, are consistent with emerging research on miRNAs, not only as biomarkers, but also as relevant regulators of disease pathophysiology and potential therapeutic targets [17, 82]. miRNA-based therapeutic strategies may include the inhibition of upregulated miRNAs (antagomiRs) or the restoration of protective miRNA activity (miRNA mimics) [83]. The potential of miRNA-based drugs is constrained by partial complementarity to target mRNAs, resulting in pleiotropic gene regulation and an increased risk of off-target effects [83]. Hence, their successful clinical translation requires advanced sequence design and precise, tissue-specific delivery, particularly in metabolically active tissues such as EAT [83]. However, the cross-sectional, case-control design of our study inherently limits inferences regarding causality or temporal sequence. Therefore, experimental validation in cellular and animal models is necessary to determine whether alterations in hsa-miR-4750-3p expression could potentially influence epicardial adipose tissue biology and, in turn, contribute to the development of diabetic coronary atherosclerosis.

Considering the heterogeneous origin of diabetic atherosclerosis, increased EAT thickness and dysregulated expression of hsa-miR-4505, hsa-miR-4743-5p, and hsa-miR-4750-3p emerged as independent determinants of CAD in T2DM after adjusting for clinical factors, with EAT thickness being the most prominent, conferring a more than fivefold increase in CAD risk. It reinforces prior evidence demonstrating that EAT accumulation predicts coronary artery atherosclerosis in T2DM, independent of conventional and imaging cardiovascular risk factors [40, 84–86]. Along the same lines, population-based studies including the Multi-Ethnic Study of Atherosclerosis and the EPICardial adipose tissue in HEART diseases study identified EAT as a standalone predictor of coronary calcification and atherosclerotic plaque burden [53, 87, 88].

To translate the complex mechanistic role of EAT into clinical practice, we evaluated its ability to detect CAD in T2DM. In our study, echocardiographic EAT thickness effectively differentiated T2DM patients with and without CAD, yielding an AUC of 0.869, with 90.0% sensitivity and 73.3% specificity at a threshold of 6.04 mm. Similar results were obtained by Uygur et al., who used MDCT-derived EAT volume as an alternative imaging approach, though they reported slightly lower efficacy in predicting coronary atherosclerosis in T2DM, with an AUC of 0.718 for a cut-off point of 5.74 mL [41]. Although data on the diagnostic value of EAT in detecting diabetes-related CAD are limited, several prior studies have indicated that EAT thickness greater than 6.05 mm may assist in identifying CAD in patients undergoing cardiac surgery, and that a value exceeding 6.1 mm was a predictor of increased carotid intima-media thickness, a recognized surrogate marker of subclinical atherosclerosis [89, 90].

**Fig. 7:**
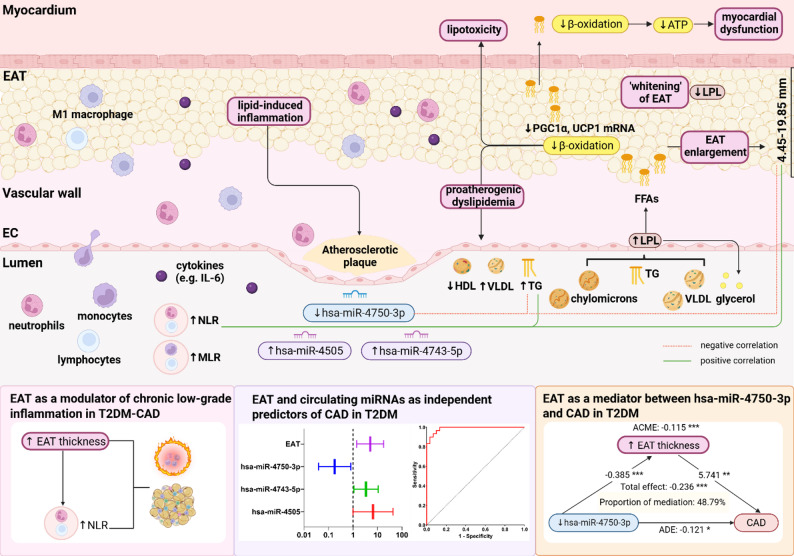
Schematic diagram of potential EAT-related mechanisms linking lipid metabolism, inflammation, and circulating miRNAs in T2DM-CAD. The concept of the illustration was based on our findings and supporting evidence from the literature data. Created with BioRender.com, accessed on 24 September 2025. T2DM-CAD, type 2 diabetes mellitus with coronary artery disease; T2DM, type 2 diabetes mellitus; CAD, coronary artery disease; EAT, epicardial adipose tissue; EC, endothelial cell; NLR, neutrophil-to-lymphocyte ratio; MLR, monocyte-to-lymphocyte ratio; IL-6, interleukin-6; TG, triglycerides; HDL, high-density lipoprotein; VLDL, very low-density lipoprotein; PGC1α, peroxisome proliferator-activated receptor gamma coactivator 1-alpha; UCP1, uncoupling protein 1; FFAs, free fatty acids; LPL, lipoprotein lipase; ATP, adenosine triphosphate; ACME, average causal mediation effect; ADE, average direct effect

In the prospective Thousand&2 cohort study of 1,030 patients with T2DM, Christensen et al. were the first to demonstrate that EAT thickness greater than 5 mm not only predicted cardiovascular events and mortality, but also enhanced risk stratification when added to existing cardiac risk algorithms [91]. Contrary to expectations, we did not show a significant gain in diagnostic performance when EAT was combined with homocysteine as clinical indicator. While hyperhomocysteinemia has been linked to mechanisms relevant to diabetic atherosclerosis, including endothelial dysfunction, pro-inflammatory activation, and pro-thrombotic effects, evidence for its independent predictive value for CAD in patients with T2DM still remains inconsistent [92]. Importantly, the addition of the previously described three-miRNA signature to the EAT thickness resulted in an excellent discrimination ability between T2DM-CAD and T2DM patients, outperforming both the EAT thickness, the clinical model, and their combination [18]. Although the improvement in classification power of the model incorporating EAT and miRNAs over the molecular model alone was not statistically significant, the lower AICc and SE values suggested its better fit and stability. Ultimately, the integration of clinical, imaging, and molecular parameters showed no increase in diagnostic performance, indicating that the parsimonious strategy combining EAT thickness and hsa-miR-4505, hsa-miR-4743-5p, hsa-miR-4750-3p expression represented the most robust approach. This supported the hypothesis that epicardial fat thickness complemented diagnostic information of diabetic atherosclerosis and highlighted its added value into a molecular framework for better CAD detection in patients with T2DM (Fig. 7).

### Limitations of the study

Several limitations of the study should be acknowledged. First, it was a single-center, cross-sectional, case-control study, thus, unavoidable selection bias and residual confounding cannot be entirely ruled out, despite the rigorous methodological approach. The study design restricts causal inference regarding the proposed mediating effect of EAT, and therefore should be framed strictly as exploratory, hypothesis-generating findings that require confirmation in prospective longitudinal studies to elucidate the potential causal mechanism. Second, the data are derived from a relatively small, single-ethnicity sample with limited subgroup sizes, which reduces the statistical power of the analysis, increasing the risk of unstable parameter estimates and false negative results. Therefore, the wide confidence interval of the mediated proportion limits the precision of the mediation estimates and should be interpreted with caution. Additionally, all of this may constrain the external validity of our results. Third, as the study included only patients with angiographically confirmed obstructive CAD, underrepresentation of individuals with intermediate stenoses may have introduced a certain degree of selection bias. Fourth, EAT thickness was measured using TTE, which assesses linear rather than volumetric fat accumulation and thus reflects regional epicardial adiposity instead of total EAT burden, limiting in-depth mechanistic interpretation. Nevertheless, echocardiographic EAT thickness demonstrates excellent correlation with epicardial fat measurements on CMR and represents a non-invasive, low-cost, easily accessible, reproducible, and clinically relevant method for EAT assessment [29, 93, 94]. Fifth, although an association between circulating miRNA and EAT thickness was observed, the lack of direct data at the tissue level precludes mechanistic conclusions. Hence, more exploratory studies are required to determine the miRNA profile and their downstream targets in EAT to elucidate their potential role in EAT-related CAD in T2DM. Last, but not least, our study did not evaluate the levels of other cytokines, adipocytokines, and markers of endothelial dysfunction, which might better reflect the complex pathogenesis of diabetic atherosclerosis, but conducting such assessments within a single study remains challenging. Moreover, the use of a limited biomarker panel in the context of a relatively small sample size may increase susceptibility to model overfitting and suggest that the predictive performance may not generalize to broader clinical settings. Further large-scale multi-center research are warranted to provide external validation of our results.

## Conclusions

In conclusion, our study demonstrated that echocardiographic EAT thickness was significantly greater in patients with T2DM and CAD compared to T2DM subjects. Among several cardiometabolic risk factors considered, only EAT, along with hsa-miR-4505, hsa-miR-4743-5p, and hsa-miR-4750-3p, served as independent contributors to CAD in T2DM. Although EAT, as a novel non-invasive imaging marker of diabetic atherosclerosis, displayed good diagnostic performance, the addition of molecular markers could improve CAD detection in T2DM. Ultimately, we proposed EAT as a candidate mediator that may partially explain the association between hsa-miR-4750-3p and diabetes-related CAD, providing a theoretical foundation with potential clinical applicability that merits further investigation in large, prospective cohort studies.

## Supplementary Information

Additional file 1: Figure S1: Bland-Altman plot showing bias and LOA between EAT thickness measurements in PLAX and PSAX views. Table S1. Intraclass correlation coefficients for intra- and interobserver variability of EAT measurements. Figure S2. Differences in EAT thickness according to sex and antidiabetic treatment used in T2DM patients with/without CAD. Figure S3. Differences in EAT thickness between obese and non-obese study participants. Table S2. Plasma miRNA expression levels in T2DM patients with/without CAD, CAD subjects, and healthy controls. Figure S4. Spearman’s correlation matrix of EAT thickness, circulating miRNAs, and clinical parameters. Figure S5. ROC curve analysis of EAT thickness. Table S3. Summary of basic parameters and standard quality measures of the models. Table S4. Comparison of the ROC curves of the models. Figure S6. Sensitivity analysis plots illustrating the model parameter ρ for checking the sequential ignorability assumption.

## Data Availability

The datasets used and/or analyzed during the current study are available from the corresponding author on reasonable request.

## Abbreviations

2D TTE: Two-dimensional transthoracic echocardiography
ACME: Average causal mediation effect
ADE: Average direct effect
ANOVA: One-way analysis of variance
AUC: Area under the curve
BMI: Body mass index
BSA: Body surface area
CAD: Coronary artery disease
CI: Confidence interval
DBP: Diastolic blood pressure
EAT: Epicardial adipose tissue
eGFR: Estimated glomerular filtration rate
FFA: Free fatty acid
FFM: Fat-free mass
FM: Fat mass
FS: Fractional shortening
HbA1c: Glycated hemoglobin A1c
HC: Hip circumference
HDL-C: High-density lipoprotein cholesterol
hs-CRP: High-sensitivity C-reactive protein
IL-6: Interleukin-6
IQR: Interquartile range
LDL-C: Low-density lipoprotein cholesterol
LV: Left ventricular
LVEDD: Left ventricular end-diastolic diameter
LVEDV: Left ventricular end-diastolic volume
LV EF: Left ventricular ejection fraction
LVESD: Left ventricular end-systolic diameter
LVESV: Left ventricular end-systolic volume
MDRD: Modification of Diet in Renal Disease
miRNA, miR: microRNA
MLR: Monocyte-to-lymphocyte ratio
NLR: Neutrophil-to-lymphocyte ratio
PLAX: Parasternal long-axis
PLR: Platelet-to-lymphocyte ratio
PSAX: Parasternal short-axis
ROC: Receiver operating characteristic
RQ: Relative quantification
RT-qPCR: Real-time polymerase chain reaction
RV: Right ventricle
SBP: Systolic blood pressure
SD: Standard deviation
T2DM: Type 2 diabetes mellitus
T2DM-CAD: Type 2 diabetes mellitus with coronary artery disease
TBW: Total body water
TC: Total cholesterol
TE: Total effect
TG: Triglycerides
WC: Waist circumference
VFR: Visceral fat rating
WHR: Waist-to-hip ratio
