# Supplementary material for: Epicardial adipose tissue mediates the association between circulating hsa-miR-4750-3p and coronary artery disease in patients with type 2 diabetes mellitus
Source: Cardiovasc Diabetol. 2026 Jan 15;25:39. doi: 10.1186/s12933-025-03055-2 (PMC12892446; doi:10.1186/s12933-025-03055-2)
Supplement: Supplementary file 1 — Additional file 1: Figure S1: Bland-Altman plot showing bias and LOA between EAT thickness measurements in PLAX and PSAX views. Table S1. Intraclass correlation coefficients for intra- and interobserver variability of EAT measurements. Figure S2. Differences in EAT thickness according to sex and antidiabetic treatment used in T2DM patients with/without CAD. Figure S3. Differences in EAT thickness between obese and non-obese study participants. Table S2. Plasma miRNA expression levels in T2DM patients with/without CAD, CAD subjects, and healthy controls. Figure S4. Spearman’s correlation matrix of EAT thickness, circulating miRNAs, and clinical parameters. Figure S5. ROC curve analysis of EAT thickness. Table S3. Summary of basic parameters and standard quality measures of the models. Table S4. Comparison of the ROC curves of the models. Figure S6. Sensitivity analysis plots illustrating the model parameter ρ for checking the sequential ignorability assumption. [file 12933_2025_3055_MOESM1_ESM.pdf]

Supplementary Material

Epicardial adipose tissue mediates the association between circulating hsa-miR-4750-3p and coronary artery disease in patients with type 2 diabetes mellitus

Joanna Szydełko [1*], Tomasz Zapolski [2], Monika Lenart-Lipińska [1], Marcin Czop [3], Alicja Petniak [3], Janusz Kocki [3] and Beata Matyjaszek-Matuszek [1]

[1] Department of Endocrinology, Diabetology and Metabolic Diseases, Medical University of Lublin, Jaczewskiego 8, 20-090 Lublin, Poland
[2] Department of Cardiology, Medical University of Lublin, Jaczewskiego 8, 20-090 Lublin, Poland
[3] Department of Clinical Genetics, Medical University of Lublin, Radziwillowska 11, 20-080 Lublin, Poland

[*] Correspondence: jszydelko@interia.pl; +48-81-72-44-668

Supplementary information content list:

Supplementary Figure 1. Bland-Altman plot showing bias and LOA between EAT thickness measurements in PLAX and PSAX views.

Supplementary Table 1. Intraclass correlation coefficients for intra- and interobserver variability of EAT measurements.

Supplementary Figure 2. Differences in EAT thickness according to sex and antidiabetic treatment used in T2DM patients with/without CAD.

Supplementary Figure 3. Differences in EAT thickness between obese and non-obese study participants.

Supplementary Table 2. Plasma miRNA expression levels in T2DM patients with/without CAD, CAD subjects, and healthy controls.

Supplementary Figure 4. Spearman's correlation matrix of EAT thickness, circulating miRNAs, and clinical parameters.

Supplementary Figure 5. ROC curve analysis of EAT thickness.

Supplementary Table 3. Summary of basic parameters and standard quality measures of the models.

Supplementary Table 4. Comparison of the ROC curves of the models.

Supplementary Figure 6. Sensitivity analysis plots illustrating the model parameter ρ for checking the sequential ignorability assumption.

Statements:

All the raw data supporting the findings in this work can be obtained on reasonable request from the corresponding author.

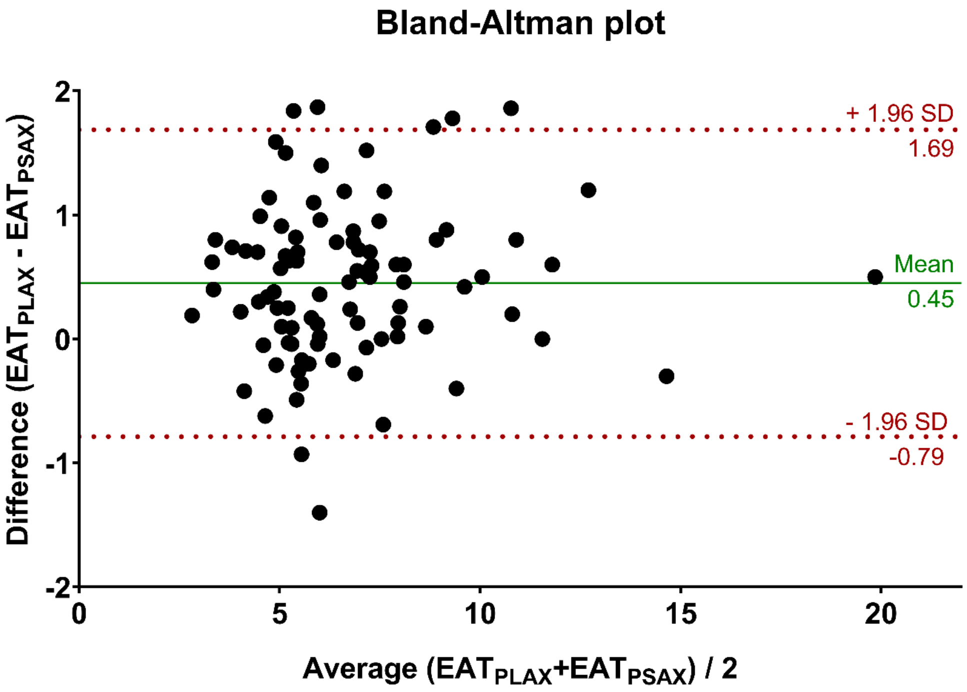

Supplementary Figure 1. Bland-Altman plot showing bias and LOA between EAT thickness measurements in PLAX and PSAX views. Data are plotted as the average of the measurements in the two projections against their difference. The mean difference (bias) is presented by the solid green line, whereas the dotted red lines indicate 95% LOA (±1.96 times the standard deviation of bias). The upper LOA is 1.69 (95% CI, 1.46 – 1.91), and the lower LOA is -0.79 (95% CI, -0.56 – -1.01). EAT, epicardial adipose tissue; PLAX, parasternal long-axis; PSAX, parasternal short-axis; SD, standard deviation; LOA, limits of agreement; CI, confidence interval

Supplementary Table 1. Intraclass correlation coefficients for intra- and interobserver variability of EAT measurements.

**Variable**	Intraobserver variability			Interobserver variability		
	**ICC**	**95% CI**	**CoV [%]**	**ICC**	**95% CI**	**CoV [%]**
EAT-PLAX [mm]	0.98	0.94–0.99	3.19	0.95	0.89–0.98	2.09
EAT-PSAX [mm]	0.98	0.95–0.99	2.91	0.95	0.88–0.98	5.34

The ICC values were calculated based on two-way mixed- or random-effects models with an absolute agreement models for single measurements. All p-values were statistically significant, with $p < 0.001$.

EAT, epicardial adipose tissue; PLAX, parasternal long-axis; PSAX, parasternal short-axis; ICC, intraclass correlation coefficient; CI, confidence interval; CoV, coefficient of variation

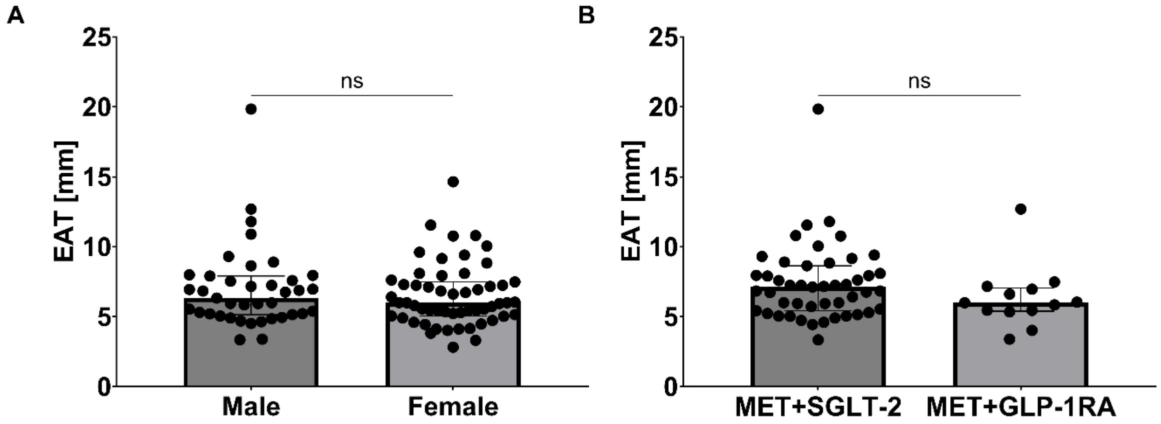

Supplementary Figure 2. Differences in EAT thickness according to sex and antidiabetic treatment used in T2DM patients with/without CAD. **(A)** There were no statistically significant differences in the median EAT thickness between men and women (6.34, IQR 5.15–7.91 vs. 6.00, IQR 5.05–7.49; $p = 0.593$) in the entire cohort ($n = 94$), as well as **(B)** the use of MET + SGLT-2 and MET + GLP-1RA (7.13, IQR 5.43–8.65 vs. 6.00, IQR 5.45–6.97; $p = 0.158$) in T2DM patients with and without CAD ($n = 60$). Data are presented as median (IQR). p-value < 0.05 was statistically significant. The analysis was performed using the Mann–Whitney *U* test. EAT, epicardial adipose tissue; MET, metformin; SGLT-2, sodium-glucose co-transporter type 2 inhibitor; GLP-1RA, glucagon-like peptide-1 receptor agonist; IQR, interquartile range

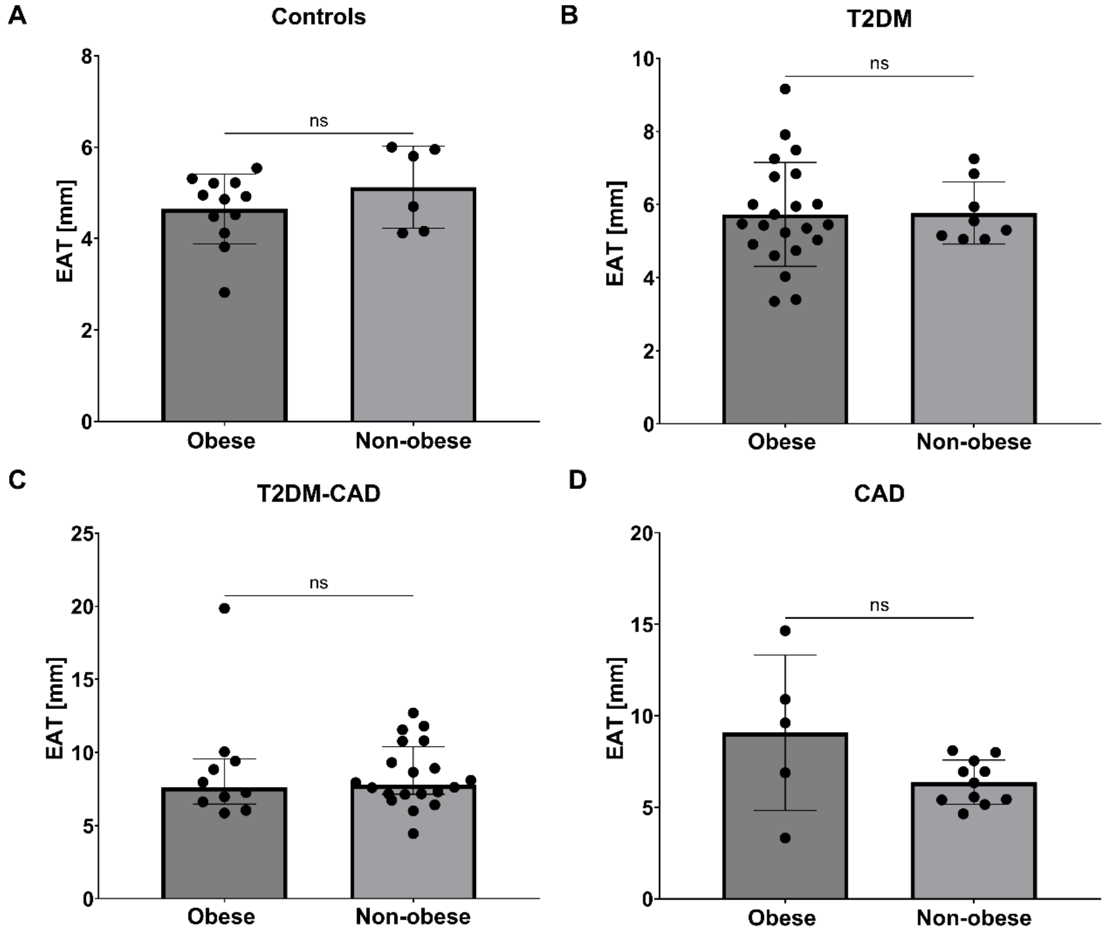

Supplementary Figure 3. Differences in EAT thickness between obese and non-obese study participants. EAT thickness was comparable between obese and non-obese **(A)** healthy controls, **(B)** T2DM, **(C)** T2DM-CAD, and **(D)** CAD patients (all $p > 0.05$). Data are presented as mean (SD) or median (IQR). p-value < 0.05 was statistically significant. The analysis was performed using Student's t-test or the Mann–Whitney U test. T2DM-CAD, type 2 diabetes mellitus with coronary artery disease; T2DM, type 2 diabetes mellitus; CAD, coronary artery disease; EAT, epicardial adipose tissue; SD, standard deviation; IQR, interquartile range

Supplementary Table 2. Plasma miRNA expression levels in T2DM patients with/without CAD, CAD subjects, and healthy controls [1].

Variable	T2DM-CAD (n = 30)	T2DM (n = 30)	CAD (n = 16)	Controls (n = 18)	p-Value
hsa-miR-4505 [$\log_{10}$RQ]	$0.73 \pm 0.59^{1,3***}$	$-0.09 \pm 0.37^{2***}$	$0.87 \pm 0.60^{1***}$	0.00 ± 0.25	$< 0.001^{***}$
hsa-miR-4743-5p [$\log_{10}$RQ]	$0.90 \pm 0.46^{1,3***}$	$0.25 \pm 0.42^{2***}$	$1.10 \pm 0.59^{1***}$	0.00 ± 0.53	$< 0.001^{***}$
hsa-miR-4750-3p [$\log_{10}$RQ]	$-0.72 \pm 0.45^{1,3***}$	$-0.18 \pm 0.35^{2*}$	$-0.66 \pm 0.37^{1***}$	0.00 ± 0.43	$< 0.001^{***}$

RT-qPCR was used to quantify miRNA expression. Data were normalized to U6 snRNA as an endogenous control, and the relative miRNA expression was logarithmically transformed to reduce skewness. Values are presented as mean ± SD.

[1] Significantly different from controls; [2] Significantly different from CAD; [3] Significantly different from T2DM. p-value < 0.05 was statistically significant. $^{*}p < 0.05$; $^{**}p < 0.01$; $^{***}p < 0.001$. The analysis was performed using one-way ANOVA with Tukey's post hoc test for unequal n.

T2DM-CAD, type 2 diabetes mellitus with coronary artery disease; T2DM, type 2 diabetes mellitus; CAD, coronary artery disease; $\log_{10}$RQ, logarithmically transformed relative expression; RT-qPCR, reverse transcription quantitative real-time polymerase chain reaction; SD, standard deviation

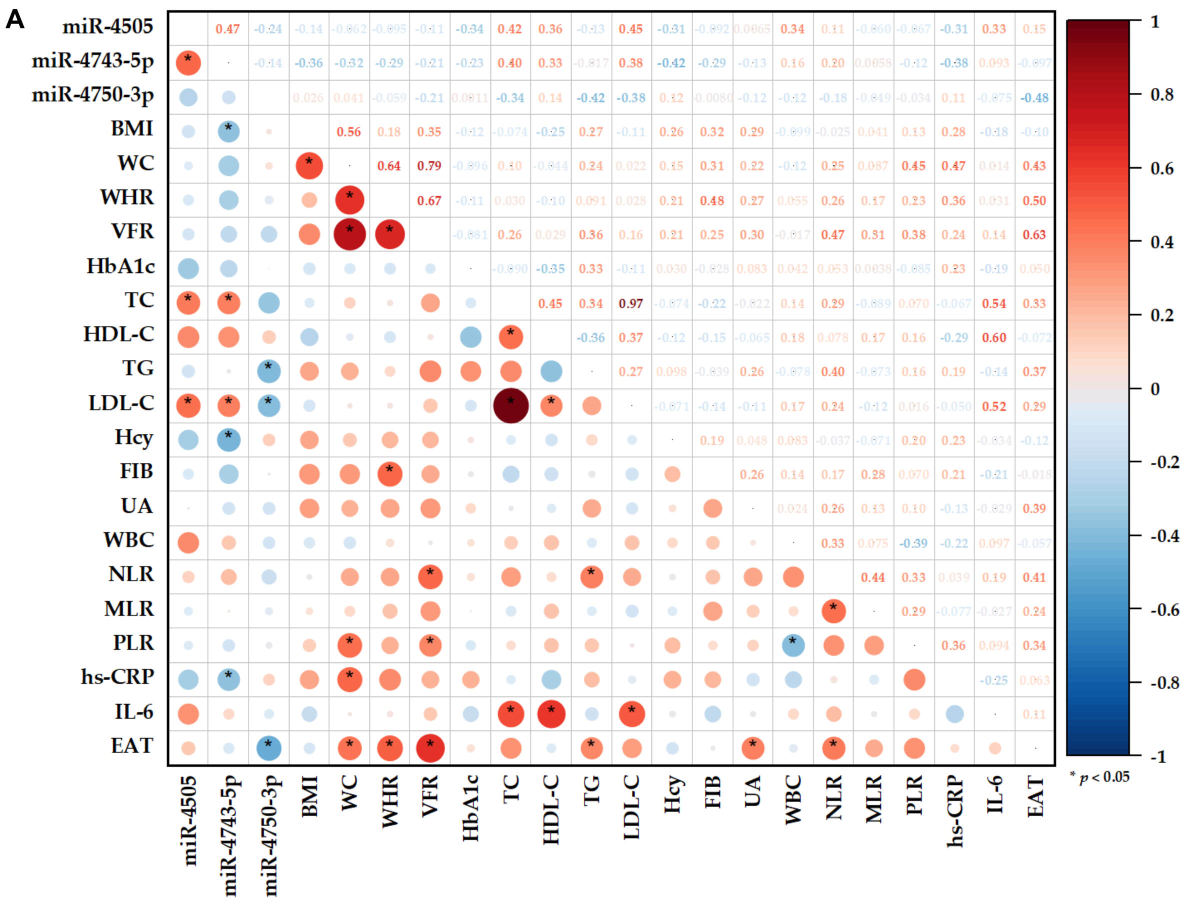

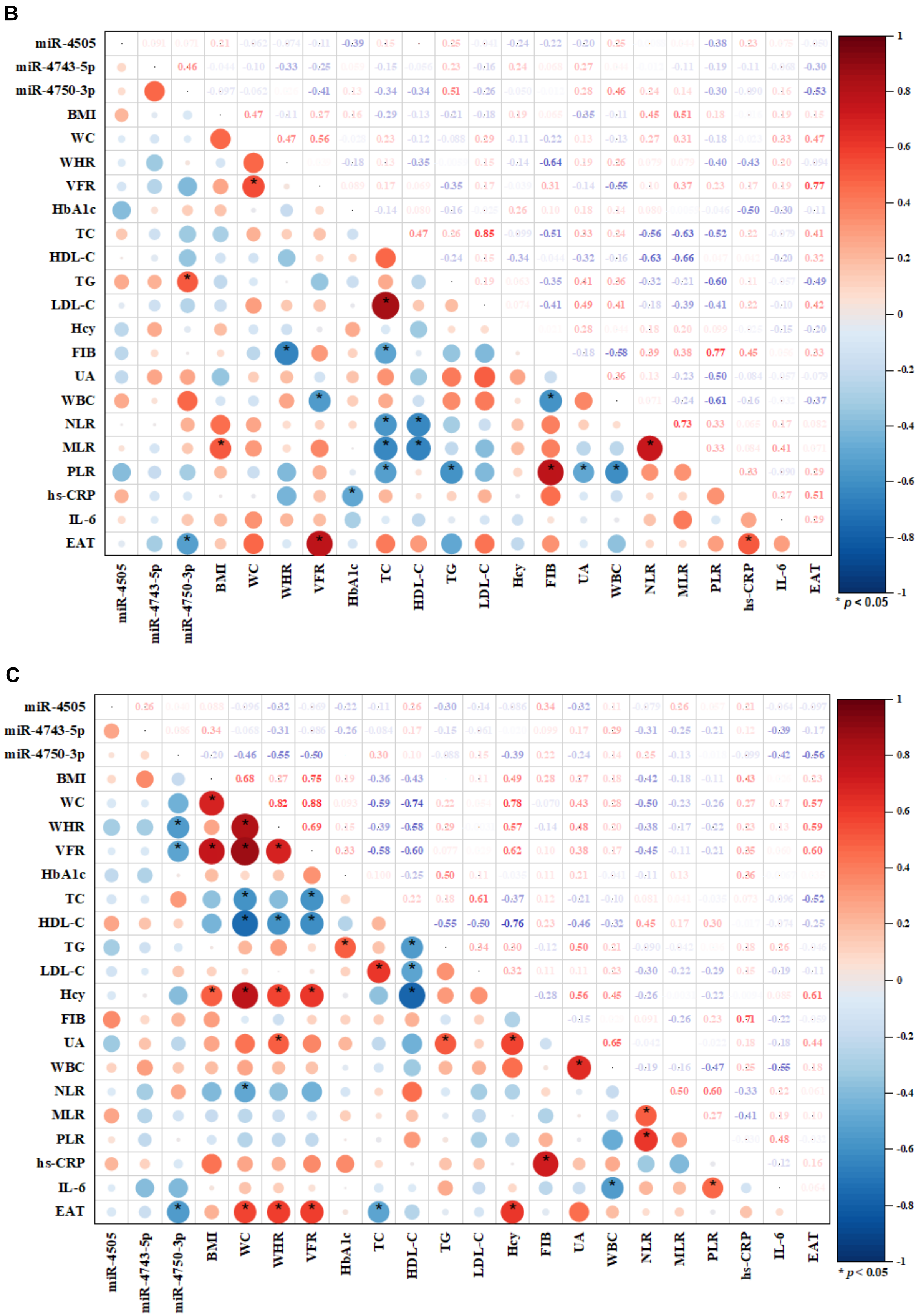
B
C
miR-4505
miR-4743-5p
miR-4750-3p
BMI
WC
WHR
VFR
HbA1c
TC
HDL-C
TG
LDL-C
Hcy
FIB
UA
WBC
NLR
MLR
PLR
hs-CRP
IL-6
EAT
1
0.8
0.6
0.4
0.2
0
-0.2
-0.4
-0.6
-0.8
-1
* p < 0.05

Supplementary Figure 4. Spearman's correlation matrix of EAT thickness, circulating miRNAs, and clinical parameters. **(A)** T2DM-CAD, **(B)** CAD, **(C)** healthy controls. The upper half of the diagonal gives the numerical value of the correlation coefficient, while the lower diagonal visualizes the correlation coefficient. The size and intensity of the circles correspond to the strength of the correlation, with larger and darker circles indicating stronger associations. Red circles represent positive correlations, while blue circles indicate negative correlations. * p-value < 0.05 was statistically significant. T2DM-CAD, type 2 diabetes mellitus with coronary artery disease; CAD; coronary artery disease; BMI, body mass index; WC, waist circumference; WHR, waist-to-hip ratio; VFR, visceral fat rating; HbA1c, glycated hemoglobin A1c; TC, total cholesterol; HDL-C, high-density lipoprotein cholesterol; TG, triglycerides; LDL-C, low-density lipoprotein cholesterol; Hcy, homocysteine; FIB, fibrinogen; UA, uric acid; WBC, white blood cell; NLR, neutrophil-to-lymphocyte ratio; MLR, monocyte-to-lymphocyte ratio; PLR, platelet-to-lymphocyte ratio; hs-CRP, high-sensitivity C-reactive protein; IL-6, interleukin 6; EAT, epicardial adipose tissue

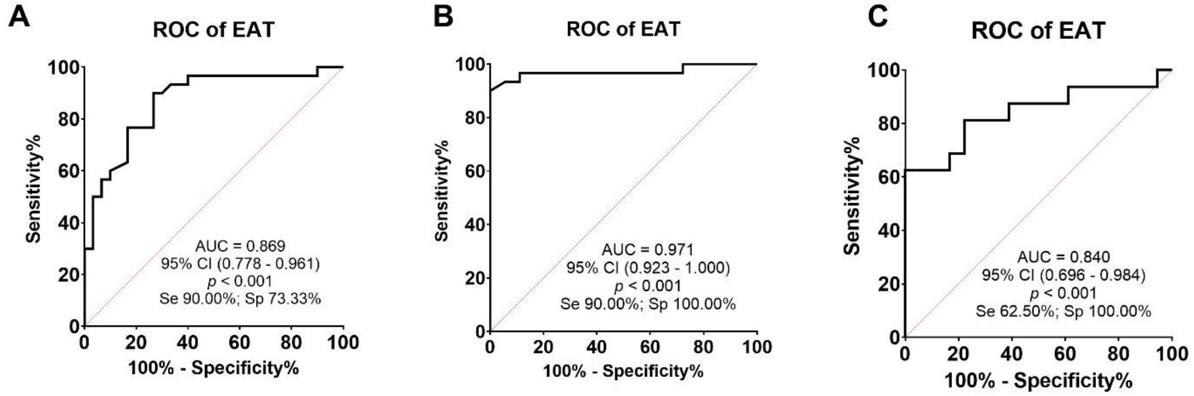

Supplementary Figure 5. ROC curve analysis of EAT thickness. **(A)** T2DM-CAD *vs.* T2DM, **(B)** T2DM-CAD *vs.* healthy controls, and **(C)** CAD *vs.* healthy controls. The AUC value, 95%CI, and level of statistical significance are indicated in each graph. p-value < 0.05 was statistically significant. T2DM-CAD, type 2 diabetes mellitus with coronary artery disease; T2DM, type 2 diabetes mellitus; CAD, coronary artery disease; EAT, epicardial adipose tissue; ROC, receiver operating characteristic; AUC, area under the curve; CI, confidence interval; Se, sensitivity; Sp, specificity

Supplementary Table 3. Summary of basic parameters and standard quality measures of the models.

Models	Variables	AUC	95% CI	*p*-Value	Se [%]	Sp [%]	PPV [%]	NPV [%]	AICc	Intercept	Coefficients
Model 1	x_1 = EAT	0.869	0.778–0.961	< 0.001	90.00	73.33	77.14	88.00	-	-	-
Model 2	x_1 = Hcy<br>x_2 = NLR<br>x_3 = hs-CRP	0.829	0.722–0.935	< 0.0001	76.67	80.00	79.31	77.42	68.91	-5.266	x_1 = 0.184<br>x_2 = 1.246<br>x_3 = -0.292
Model 3	x_1 = EAT<br>x_2 = Hcy	0.901	0.826–0.976	< 0.0001	96.67	73.33	78.38	95.65	50.98	-11.104	x_1 = 1.137<br>x_2 = 0.181
Model 4 and 5	x_1 = EAT<br>x_2 = hsa-miR-4505<br>x_3 = hsa-miR-4743-5p<br>x_4 = hsa-miR-4750-3p	0.988	0.970–1.000	< 0.0001	100.00	86.67	88.24	100.00	25.30	-15.925	x_1 = 1.615<br>x_2 = 6.247<br>x_3 = 4.049<br>x_4 = -5.729

AUC, area under the curve; CI, confidence interval; Se, sensitivity; Sp, specificity; PPV, positive predictive value; NPV, negative predictive value; AICc, corrected Akaike information criterion; EAT, epicardial adipose tissue; Hcy, homocysteine; NLR, neutrophil-to-lymphocyte ratio; hs-CRP, high-sensitivity C-reactive protein

Supplementary Table 4. Comparison of the ROC curves of the models.

Variable	Combined AUC = 0.998	EAT + Molecular AUC = 0.998	EAT + Clinical AUC = 0.901	Clinical AUC = 0.829	EAT AUC = 0.869
**Combined**	Combined				
**EAT + Molecular**	1.000	EAT + Molecular			
**EAT + Clinical**	0.026 *	0.026 *	EAT+ Clinical		
**Clinical**	0.004 *	0.004 *	0.282	Clinical	
**EAT**	0.013 *	0.013 *	0.597	0.586	EAT

p-value < 0.05 was statistically significant. The difference in area under the ROC curves of the models was calculated using the nonparametric DeLong's test.
ROC, receiver operating characteristic; AUC, area under the curve; EAT, epicardial adipose tissue

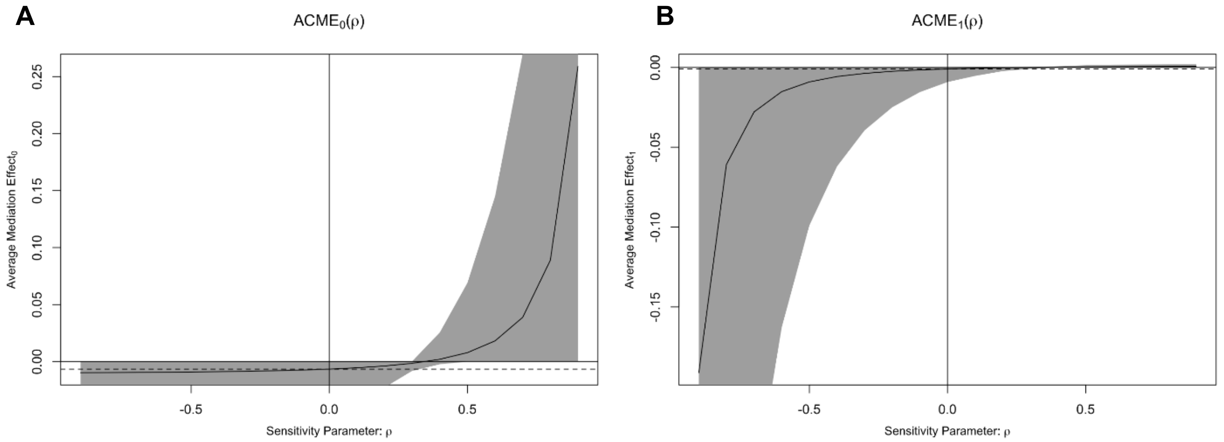

Supplementary Figure 6. Sensitivity analysis plots illustrating the model parameter ρ for checking the sequential ignorability assumption. **(A)** T2DM group (ρ = 0.30), **(B)** T2DM-CAD group (ρ = 0.30). The plots show the corresponding change in ACME resulting from changing the sensitivity parameter value (ρ). The solid line and grey area represent the estimate and 95% CIs corresponding to the ACME. The dashed line indicates the specific ρ values at which the ACME approaches zero. ACME, average causal mediation effect; T2DM, type 2 diabetes mellitus; T2DM-CAD, type 2 diabetes mellitus with coronary artery disease; CI, confidence interval

References:

1. Szydełko J, Czop M, Petniak A, Lenart-Lipińska M, Kocki J, Zapolski T, et al. Identification of plasma miR-4505, miR-4743-5p and miR-4750-3p as novel diagnostic biomarkers for coronary artery disease in patients with type 2 diabetes mellitus: a case-control study. Cardiovasc Diabetol. 2024;23:278.
